# Synergistic Cu–OH and zeolite confinement in Cu/MCM-22 for benzaldehyde-mediated Mukaiyama epoxidation of long-chain α-olefins

**DOI:** 10.1093/nsr/nwaf502

**Published:** 2025-11-14

**Authors:** Hongying Chang, Ziyu Zhou, Peng He, Kun Zhang, Yujie Xie, Xiangjie Zhang, Tao Yan, Min Zhang, Gaolei Qin, Huaming Hou, Yanyan Chen, Jianguo Wang, Zhi Cao

**Affiliations:** School of Chemical Sciences, University of Chinese Academy of Sciences, Beijing 100190, China; National Energy Center for Coal to Clean Fuels, Synfuels China Technology Co., Ltd., Beijing 101407, China; School of Chemical Sciences, University of Chinese Academy of Sciences, Beijing 100190, China; State Key Laboratory of Coal Conversion, Institute of Coal Chemistry, Chinese Academy of Sciences, Taiyuan 030001, China; National Energy Center for Coal to Clean Fuels, Synfuels China Technology Co., Ltd., Beijing 101407, China; State Key Laboratory of Coal Conversion, Institute of Coal Chemistry, Chinese Academy of Sciences, Taiyuan 030001, China; National Energy Center for Coal to Clean Fuels, Synfuels China Technology Co., Ltd., Beijing 101407, China; School of Chemical Sciences, University of Chinese Academy of Sciences, Beijing 100190, China; State Key Laboratory of Coal Conversion, Institute of Coal Chemistry, Chinese Academy of Sciences, Taiyuan 030001, China; National Energy Center for Coal to Clean Fuels, Synfuels China Technology Co., Ltd., Beijing 101407, China; School of Chemical Sciences, University of Chinese Academy of Sciences, Beijing 100190, China; State Key Laboratory of Coal Conversion, Institute of Coal Chemistry, Chinese Academy of Sciences, Taiyuan 030001, China; National Energy Center for Coal to Clean Fuels, Synfuels China Technology Co., Ltd., Beijing 101407, China; School of Chemical Sciences, University of Chinese Academy of Sciences, Beijing 100190, China; State Key Laboratory of Coal Conversion, Institute of Coal Chemistry, Chinese Academy of Sciences, Taiyuan 030001, China; National Energy Center for Coal to Clean Fuels, Synfuels China Technology Co., Ltd., Beijing 101407, China; School of Chemical Sciences, University of Chinese Academy of Sciences, Beijing 100190, China; State Key Laboratory of Coal Conversion, Institute of Coal Chemistry, Chinese Academy of Sciences, Taiyuan 030001, China; National Energy Center for Coal to Clean Fuels, Synfuels China Technology Co., Ltd., Beijing 101407, China; School of Chemical Sciences, University of Chinese Academy of Sciences, Beijing 100190, China; State Key Laboratory of Coal Conversion, Institute of Coal Chemistry, Chinese Academy of Sciences, Taiyuan 030001, China; National Energy Center for Coal to Clean Fuels, Synfuels China Technology Co., Ltd., Beijing 101407, China; School of Chemical Sciences, University of Chinese Academy of Sciences, Beijing 100190, China; State Key Laboratory of Coal Conversion, Institute of Coal Chemistry, Chinese Academy of Sciences, Taiyuan 030001, China; National Energy Center for Coal to Clean Fuels, Synfuels China Technology Co., Ltd., Beijing 101407, China; National Energy Center for Coal to Clean Fuels, Synfuels China Technology Co., Ltd., Beijing 101407, China; State Key Laboratory of Coal Conversion, Institute of Coal Chemistry, Chinese Academy of Sciences, Taiyuan 030001, China; School of Chemical Sciences, University of Chinese Academy of Sciences, Beijing 100190, China; State Key Laboratory of Coal Conversion, Institute of Coal Chemistry, Chinese Academy of Sciences, Taiyuan 030001, China; National Energy Center for Coal to Clean Fuels, Synfuels China Technology Co., Ltd., Beijing 101407, China

**Keywords:** long-chain α-olefins, Mukaiyama epoxidation, zeolite, confinement effect, Cu–OH

## Abstract

The Mukaiyama epoxidation of olefins, leveraging molecular oxygen and aldehydes to generate epoxides, is a cornerstone of sustainable synthesis but is hindered by compromising epoxide selectivity and aldehyde coupling efficiency due to the high reactivity of acylperoxy radicals. Here, we report a Cu/MCM-22 catalyst that synergistically integrates Cu–OH active sites with zeolite confinement to achieve exceptional selectivity and efficiency in the aerobic epoxidation of long-chain linear α-olefins. Comprehensive characterization, including transmission electron microscopy, X-ray absorption spectroscopy and Fourier-transform infrared spectroscopy, confirms atomically dispersed Cu^2+^ ions within MCM-22’s framework, enabling benzaldehyde activation via Cu^2+^/Cu^+^ redox cycling and H_2_O formation. Electron paramagnetic resonance and density functional theory (DFT) studies reveal that the confined pores of MCM-22 stabilize acylperoxy radicals, suppressing undesired pathways and promoting epoxide formation. Catalytic evaluations demonstrate 97% conversion and 90% selectivity for 1-undecene epoxidation, which was further improved to 99% selectivity by deactivating the Cu sites on the zeolite external surface, resulting in a roughly 3-fold increase in aldehyde coupling efficiency and significantly outperforming unconfined Cu/Al_2_O_3_ and Cu/SiO_2_. Kinetic analyses and DFT calculations highlight reduced energy barriers (84 kJ·mol^−1^) for benzaldehyde activation and enhanced chemoselectivity driven by zeolite confinement. This work elucidates the pivotal role of tailored active sites and spatial constraints in radical catalysis for selective epoxide synthesis.

## Teaser

An innovative strategy for Mukaiyama epoxidation is developed via a Cu/MCM-22 catalyst integrating atomically isolated Cu2+ ions with zeolite confinement, unraveling how Cu2+/Cu+ redox cycling enables benzaldehyde activation.

## INTRODUCTION

**Scheme 1.**
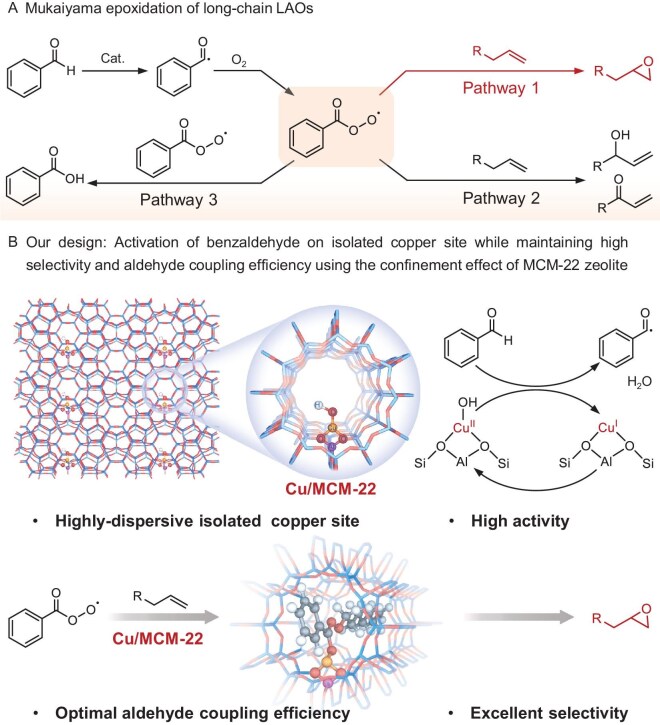
Schematic illustration of the Mukaiyama epoxidation of long-chain LAOs and our proposed catalytic system. (A) The reaction route for Mukaiyama epoxidation with benzaldehyde as the sacrificial reductant. (B) Our strategies: Cu zeolite catalyst activates aldehyde-initiated free radical reactions to increase reactivity and study the reaction mechanism. The confined zeolite channel enhances selectivity by restricting intermediate formation.

The epoxidation of olefins to epoxides is a cornerstone of industrial chemistry, as epoxides serve as vital intermediates in the synthesis of pharmaceuticals, polymers and epoxy resins [1–3]. Direct aerobic epoxidation, employing molecular oxygen (O_2_) as the oxidant, is particularly appealing due to its abundant availability and low cost [4–6]. However, except for ethylene epoxidation using O_2_ over an Ag/Al_2_O_3_ catalyst, achieving epoxidation of other olefins with O_2_ as the sole oxidant remains challenging [7]. This is primarily because converting O_2_ into epoxidation-active species is inherently difficult. To address this issue, it is necessary to use an efficient sacrificial reductant as the co-oxidation substrate in epoxidation. Notably, the Mukaiyama epoxidation, which utilizes aldehydes as sacrificial agents, is particularly prominent [8–10]; activation of the aldehyde generates acyl radicals, which then react with O_2_ to form highly active acylperoxy radicals [11–14] (Scheme 1A and Scheme S1). Yet, the pronounced reactivity of acylperoxy radicals poses a formidable challenge, often compromising epoxide selectivity [15,16] and aldehyde coupling efficiency [7,17–20] (defined as the molar ratio of epoxide produced to aldehyde consumed)—a persistent obstacle in advancing catalytic Mukaiyama epoxidation at the forefront of research [21–25].

Recently, our research group has achieved significant progress in leveraging zeolite pore confinement to modulate reactive intermediates, enabling exceptional (regio)selectivity in olefin hydroformylation reactions [26,27]. This approach has recently been extended to Mukaiyama epoxidation, where we developed a heterogeneous beta zeolite (Beta) catalyst that exploits spatial confinement within its framework to govern intermediates in competing epoxidation pathways [28]. This strategy yielded an unprecedented epoxide selectivity exceeding 95%, coupled with a 3-fold increase in aldehyde coupling efficiency. In that study, isobutyraldehyde, an aliphatic aldehyde, was employed as the reductant, readily activated by Lewis acid sites within the zeolite. However, acyl radical decarbonylation diminished aldehyde coupling efficiency and epoxide yields [28,29]. Conversely, aromatic aldehydes, stabilized by π-conjugation between the phenyl ring and carbonyl group, resist decarbonylation but are less reactive on zeolite Lewis acid sites, typically requiring redox-active metals for effective activation [30].

Drawing inspiration from homogeneous catalysis, copper(II) has emerged as a potent catalyst for oxidation reactions [31], including aldehyde and ketone oxidation [32,33], with copper-hydroxyl (Cu–OH) species exhibiting exceptional efficacy in aldehyde activation [34,35]. Building on this insight, we proposed integrating Cu–OH groups onto the inner pore surfaces of zeolites to enhance aromatic aldehyde activation, thus accelerating the rate-limiting step in Mukaiyama epoxidation [12,13]. Concurrently, by leveraging our expertise in zeolite confinement, we sought to ensure high epoxide selectivity and improved aldehyde coupling efficiency [26–28].

In this study, we utilized benzaldehyde as the sacrificial reductant to drive epoxidation and conducted a meticulous mechanistic investigation into the role of Cu–OH sites in activating benzaldehyde within spatially constrained zeolite systems (Scheme 1B). MCM-22 was chosen as the zeolite support, as it effectively exerts a confinement effect on the intermediates [36] (Table S1). Cu–OH active sites were incorporated into MCM-22 through a rigorously optimized ion-exchange method [37]. Comprehensive characterization, employing transmission electron microscopy (TEM), X-ray absorption spectroscopy (XAS) and Fourier-transform infrared (FTIR) spectroscopy, confirmed the presence of isolated Cu–OH species within the MCM-22 framework. Insights from electron paramagnetic resonance (EPR) spectroscopy, combined with experimental data and density functional theory (DFT) calculations, revealed that Cu–OH sites enable aldehyde activation via Cu^2+^/Cu^+^ redox cycling, accompanied by H_2_O formation. This process triggers radical generation, facilitating efficient olefin epoxidation. Furthermore, we assessed the substrate scope and operational stability of the Cu/MCM-22 catalyst, demonstrating its robust performance in selectively epoxidizing long-chain alkenes, thus highlighting its potential for industrial applications. This work underscores the synergistic interplay of zeolite confinement and tailored active sites in surmounting longstanding challenges in Mukaiyama epoxidation, charting a path toward highly selective and efficient catalytic systems for sustainable epoxide synthesis.

## RESULTS AND DISCUSSION

### Catalyst synthesis and characterization

Tri-coordinated Al sites, known to catalyze Mukaiyama epoxidation, may contribute activity that overlaps with extra-framework Cu sites anchored at Brønsted acid sites induced by tetra-coordinated Al species. To evaluate the feasibility of extra-framework Cu as active sites for Mukaiyama epoxidation, we selected a zeolite with moderate tri-coordinated Al activity. A series of Na-type silicoaluminate zeolites, including MCM-22, Beta and zeolite Y (Y), were assessed for 1-undecene epoxidation using O_2_ with benzaldehyde as the co-reductant. As shown in Table S2, Beta showed the highest conversion of 1-undecene, but Y and MCM-22 performed poorly, reflecting the weaker Lewis acid activity of their tri-coordinated Al sites within the zeolite framework [28,38]. Furthermore, the enhanced selectivity of MCM-22 originated from its unique pore topology, composed of 10-membered ring sinusoidal channels interlinked with 12-membered ring supercages [36]. This hierarchical architecture provided a well-confined microenvironment that matched the dimensions of key reaction intermediates, thereby promoting the desired epoxidation pathway while effectively suppressing undesired side reactions. Upon Cu incorporation, the distinctive pore system offered preferential anchoring sites that facilitated the formation of highly dispersed Cu species [39,40], leading to significantly enhanced catalytic activity compared with Cu/Beta and Cu/Y (Fig. S1, Table S3). Consequently, attention was focused on Cu/MCM-22 to explore the underlying mechanism responsible for the Cu-induced activity improvement in Mukaiyama epoxidation.

The Cu/MCM-22 catalyst was synthesized via ion exchange of Na-type MCM-22 (Si/Al = 12.5) in an aqueous Cu(NO_3_)_2_ solution, with the pH maintained between 4.5 and 5.5 to prevent Cu^2+^ precipitation and ensure substitution of Na^+^ at Brønsted acid sites. For comparison, Cu/Al_2_O_3_ and Cu/SiO_2_ were prepared using an analogous method. Powder X-ray diffraction (XRD) confirmed that Cu/MCM-22 retained its MWW-type topology without crystalline CuO or Cu_2_O phases [39] (Fig. S2), indicating uniform Cu dispersion. Sharp, intense diffraction peaks underscored the high crystallinity of MCM-22. Similarly, Cu/Al_2_O_3_ and Cu/SiO_2_ lacked crystalline CuO or Cu_2_O phases, suggesting well-dispersed Cu on these open-structured supports. N_2_ sorption analysis verified the microporous structure of Cu/MCM-22 (Fig. S3 and Table S4), with Brunauer–Emmett–Teller (BET) results revealing average pore sizes of 6.1 nm for Cu/Al_2_O_3_ and 24.0 nm for Cu/SiO_2_, far larger than the microporous size (0.5 nm) of MCM-22 (Table S4). Inductively coupled plasma (ICP) analysis determined a Cu content of 0.5 wt% in Cu/MCM-22, comparable to Cu/Al_2_O_3_ and Cu/SiO_2_, corresponding to a Cu/Al ratio of 0.08 (Table S4). Scanning electron microscopy (SEM) revealed the rose-like morphology of Cu/MCM-22, comprising loosely aggregated disc-shaped particles (∼800 nm) composed of 15–25 nm thick slices (Fig. S4). In contrast, Cu/Al_2_O_3_ and Cu/SiO_2_ particles were ∼100 nm (Fig. S5).

**Figure 1.**
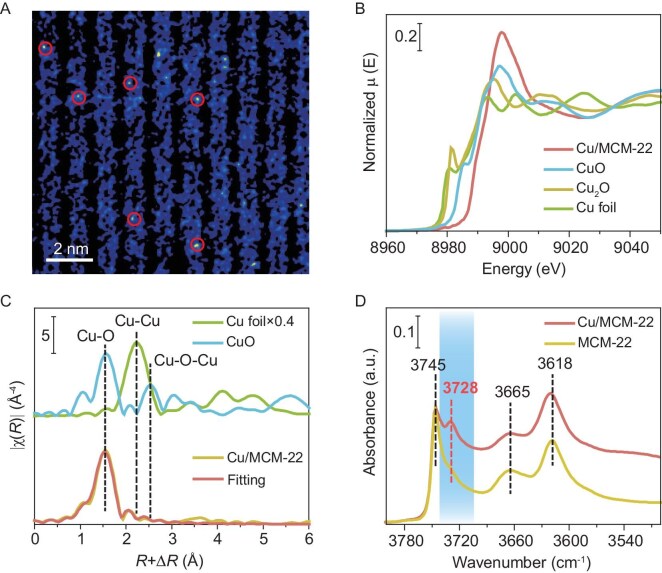
Structural and coordination environment characterization of Cu/MCM-22. (A) Aberration-corrected HAADF-STEM image of Cu/MCM-22, highlighting atomically dispersed Cu atoms with red circles. (B) Normalized XANES and (C) EXAFS spectra with curve-fitting at the Cu K-edge for Cu/MCM-22. (D) FTIR spectra in the OH stretching region for Cu/MCM-22 and MCM-22.

To probe the local environment of Cu in MCM-22, TEM images showed no Cu clusters (Figs S6 and S7), with energy-dispersive X-ray spectroscopy (EDS) confirming homogeneous Cu distribution across MCM-22 crystallites (Fig. S8). Aberration-corrected high-angle annular dark-field scanning transmission electron microscopy (HAADF-STEM) revealed isolated bright spots (marked by red circles), indicating single-site Cu species in Cu/MCM-22 (Fig. 1A). Conversely, Cu/Al_2_O_3_ and Cu/SiO_2_ displayed 1.6 nm CuO nanoparticles (Fig. S9), suggesting that Al_2_O_3_ and SiO_2_ cannot stabilize highly dispersed Cu. X-ray photoelectron spectroscopy (XPS) identified Cu(II) in Cu/MCM-22 via Cu 2p_3/2_ and 2p_1/2_ binding energy peaks and satellites (Fig. S10), corroborated by Cu LMM Auger electron spectroscopy (AES) peaks at 572.5 eV [41] (Fig. S11). XAS, including X-ray absorption near-edge structure (XANES), showed a white line position for Cu/MCM-22 exceeding that of CuO, consistent with single-site Cu(II) systems (Fig. 1B). The Cu K-edge extended X-ray absorption fine structure (EXAFS) spectrum exhibited a peak at 1.53 Å (Cu–O bonding) without peaks at 2.23 Å (Cu–Cu) or 2.54 Å (Cu–O–Cu), confirming the absence of Cu clusters [41,42] (Fig. 1C and Fig. S12) FTIR spectroscopy of Na-type MCM-22 displayed peaks at 3745, 3665 and 3618 cm^−1^, attributed to Si–OH, Al–OH and Si–OH–Al groups [43], respectively. Cu incorporation introduced a peak at 3728 cm^−1^, linked to OH vibrations associated with Cu species [44,45] (Fig. 1D). Collectively, HAADF-STEM, XAS and FTIR data indicate that Cu(II) atoms in Cu/MCM-22 are atomically dispersed, coordinated with oxygen atoms at Brønsted acid sites and hydroxyl groups for charge balance.

### Catalytic performance evaluation

**Figure 2.**
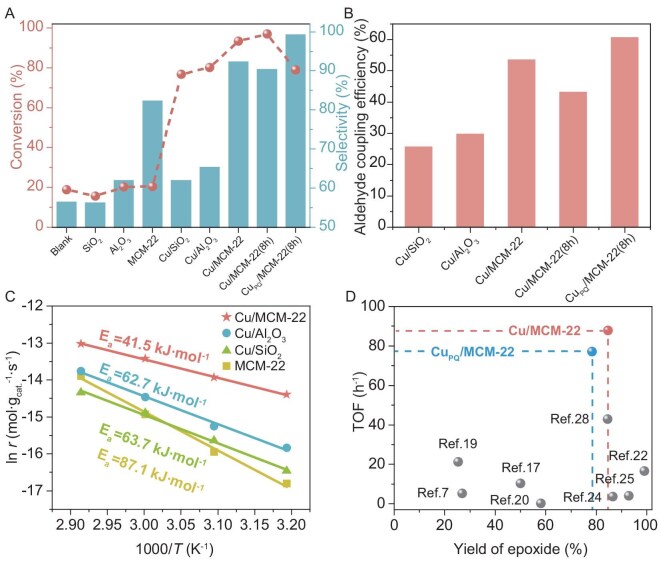
Catalytic evaluation of various catalysts. (A) Conversion of 1-undecene and selectivity for 1,2-epoxyundecane catalyzed by unconfined and confined zeolite systems (see Tables S6 and S8 for details). (B) Benzaldehyde coupling efficiency for various catalysts, defined as the molar amount of epoxide as a percentage of the molar amount of consumed aldehyde, simultaneously (see Tables S6 and S8 for details). (C) Arrhenius plots for the epoxidation of 1-undecene catalyzed by Cu/MCM-22, Cu/Al_2_O_3_, Cu/SiO_2_ and MCM-22. (D) Comparison of the catalytic performances of Cu/MCM-22 and Cu_PQ_/MCM-22 with previously reported catalysts in LAO epoxidation (see Table S11 for details).

Control experiments elucidated the influence of reaction conditions on olefin conversion and epoxide selectivity (Figs S13–S17). Optimal conditions, determined as 60°C, 6 h, 600 rpm, 5.0 mL of acetonitrile and a benzaldehyde:1-undecene molar ratio of 5:1 (see details in Figs S13–S17), were adopted for subsequent studies. When air was substituted for pure O_2_, conversion of 1-undecene dropped significantly to 21.6% (Table S5), demonstrating that a high O_2_ concentration is critical for effective epoxidation. The conversion of 1-undecene and selectivity for 1,2-epoxyundecane are shown in Fig. 2A and Table S6. Without a catalyst, 1-undecene conversion was limited to 18.9%, with 56.3% selectivity for 1,2-epoxyundecane. SiO_2_ as a catalyst yielded negligible improvement, underscoring its inability to facilitate epoxidation effectively. Conversion rose to ∼20% with Al_2_O_3_ and Na-type MCM-22, attributed to Al species, with MCM-22 achieving a selectivity of 82.2% versus 61.9% for Al_2_O_3_, reflecting suppressed byproduct formation within zeolite channels. Incorporation of Cu species increased conversion to 76.8% for Cu/SiO_2_ and 80.1% for Cu/Al_2_O_3_, confirming the catalytic role of Cu. Remarkably, Cu/MCM-22 elevated 1-undecene conversion from 20.5% to 93.3%, highlighting the superior activity of isolated Cu–OH sites compared to Cu clusters. In addition, Cu species were incorporated into the MCM-22 zeolite via a wet impregnation method (denoted as Cu/MCM-22-Im). However, the infrared spectra revealed the absence of Cu–OH structural features in this sample (Fig. S18), and the 1-undecene conversion (83.8%) was lower than that of Cu/MCM-22 (Table S7). These results further substantiated that isolated Cu–OH species were essential for high epoxidation efficiency.

To further explore the potential of Cu/MCM-22, the reaction was extended to 8 h, achieving 97.0% 1-undecene conversion and 90.3% 1,2-epoxyundecane selectivity (Fig. 2A and Table S8), with a carbon balance exceeding 98% (Table S9). The disc-like morphology of MCM-22, with significant external surface area relative to its confined internal pores, implies that some Cu atoms, introduced via ion exchange, reside on external surfaces. These external Cu sites, unconfined by zeolite channels, may initiate epoxidation pathways leading to byproducts [28]. To test this hypothesis, we prepared Cu/MCM-22 catalysts with external Cu sites poisoned by triphenylamine (TPA) or 2-phenylquinoline (PQ), denoted Cu_TPA_/MCM-22 and Cu_PQ_/MCM-22. These catalysts reduced conversion to ∼79% over 8 h but increased selectivity to >99% (Fig. 2A and Table S8), confirming that external Cu sites contribute to byproduct formation.

Beyond selectivity, Cu/MCM-22 markedly improved benzaldehyde coupling efficiency to 53.4%, compared to 29.6% for Cu/Al_2_O_3_ and 25.6% for Cu/SiO_2_, reaching 60.5% after poisoning (Fig. 2B, Table S6 and Table S8). This enhancement suggests that zeolite confinement suppresses self-termination, enabling efficient aldehyde activation and conversion to acylperoxy radicals for olefin epoxidation. Notably, benzaldehyde byproducts were <6%, significantly lower than those reported for isobutyraldehyde [28] (Table S10).

Cu/MCM-22 also exhibited exceptional catalytic activity, supported by kinetic studies. Arrhenius plots for Mukaiyama epoxidation over MCM-22, Cu/SiO_2_, Cu/Al_2_O_3_ and Cu/MCM-22 (Fig. 2C) revealed an activation energy of 41.5 kJ mol^−1^ for Cu/MCM-22, substantially lower than the 62.7 kJ mol^−1^ for Cu/Al_2_O_3_, 63.7 kJ mol^−1^ for Cu/SiO_2_ and 87.1 kJ mol^−1^ for MCM-22, underscoring the kinetic advantage of Cu/MCM-22. Overall, these results demonstrate that Cu/MCM-22 exhibits superior catalytic performance and metal utilization in long-chain α-olefin (LAO) epoxidation compared to previously reported catalysts (Fig. 2D and Table S11). Recycling experiments demonstrated robust stability, with 1-undecene conversion and 1,2-epoxyundecane selectivity remaining consistent over six cycles (Fig. S19). XRD and N_2_ adsorption–desorption analyses of the spent catalyst confirmed unchanged textural properties (Figs S20 and S21). And the characteristic Cu–OH band at 3728 cm^−1^ remained essentially unchanged after multiple catalytic cycles (Fig. S22), indicating that the isolated Cu–OH species preserved their structural integrity and sustained catalytic activity. Besides this, the versatility of Cu/MCM-22 was further evidenced by efficient epoxidation of C_7_–C_11_ linear LAOs, achieving high conversion and selectivity (Table S12), establishing Cu/MCM-22 as a broadly applicable catalyst for LAO epoxidation. In addition to linear α-olefins, cyclooctene was also employed as a bulky olefin substrate. The result (Table S12) revealed that both the activity and selectivity of the reaction were unsatisfactory, indicating that the cyclooctene struggles to diffuse into the internal pores, which further verified the confinement effect of channel.

### Mechanistic studies

The Mukaiyama epoxidation reaction is initiated by aldehyde activation to form acyl radicals. In the proposed pathway (Scheme 1A), aldehydes undergo hydrogen atom abstraction to yield acyl radicals, which react with O_2_ to form acylperoxy radicals, the primary oxidants for epoxidation [11]. To probe the radical species involved in 1-undecene epoxidation over Cu/MCM-22, EPR spin-trapping experiments were conducted under reactive conditions, using *N-tert*-butyl-α-phenylnitrone (PBN) as the spin trap. This approach allows for the identification of transient radicals by forming more stable PBN–radical adducts. Under anaerobic conditions, a three-line EPR signal was observed with hyperfine coupling constants of *A^N^* = 14.7 G and *A^H^* = 3.7 G, which is attributed to a PBN–acyl radical adduct. Notably, the hyperfine splitting from *A^H^* is not clearly resolved due to signal broadening. Under aerobic conditions, a six-line signal with *A^N^* = 13.7 G and *A^H^* = 1.8 G was detected, characteristic of a PBN–acylperoxy radical adduct [33,46–48] (Fig. S23 and Table S13). Clear EPR signals for both radicals confirmed their pivotal roles as key intermediates driving epoxidation.

The critical role of radicals was substantiated by performing a radical quenching experiment using 2,6-ditert-butyl-4-methylphenol (BHT) as the scavenger. Without BHT, 1-undecene conversion progressively increased to 20.0%, 60.4%, 93.3%, 97.0% and 98.4% at 2, 4, 6, 8 and 10 h, respectively (Fig. S24). BHT addition completely inhibited the reaction, halting conversion (Fig. S24 and Table S14), affirming the radical-dependent nature of epoxidation [49]. As acylperoxy radicals can form peracids via hydrogen abstraction, we investigated whether peracids contribute as oxidants. Adding BHT at 2 h and extending the reaction to 10 h yielded a conversion of 21.4%, comparable to the 2 h control, indicating no further progress (Table S14). This suggests acylperoxy radicals, not peracids, are the primary oxidants, reinforcing the proposed pathway.

**Figure 3.**
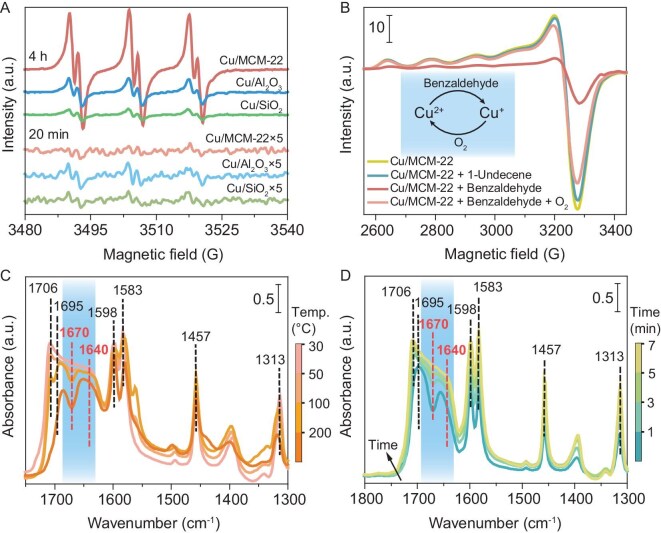
Mechanism exploration: identification of radicals and the role of Cu. (A) EPR spin-trapping spectra of acylperoxy radicals captured by PBN after 20 min and 4 h of benzaldehyde oxidation, along with the dependence of acylperoxy radical intensities on reaction time over various catalysts. (B) *Operando* EPR spectra of Cu/MCM-22 in acetonitrile solution under 100 K. FTIR spectra of Cu/MCM-22 upon benzaldehyde adsorption with respect to desorption temperature (C) and adsorption time (D).

The confinement effect of MCM-22 on intermediates was assessed by monitoring acylperoxy radical concentrations via EPR spectroscopy. After 20 min, EPR signals for acylperoxy radicals were stronger for Cu/SiO_2_ and Cu/Al_2_O_3_ than for Cu/MCM-22 (Fig. 3A). By 4 h, signals weakened for Cu/SiO_2_ and Cu/Al_2_O_3_ but intensified for Cu/MCM-22. This indicates that the channels of MCM-22 initially restrict benzaldehyde diffusion to Cu sites, moderating radical formation, unlike the unrestricted diffusion on Cu/SiO_2_ and Cu/Al_2_O_3_. The rapid activation of benzaldehyde by copper species supported on unconfined materials generates acylperoxy radicals that undergo fast self-termination, ultimately reducing aldehyde coupling efficiency [13]. The confined cages of MCM-22 spatially isolate acylperoxy radicals, suppressing autoxidation and enhancing coupling efficiency (Fig. 2B).

*Operando* EPR spectroscopy tracked Cu species evolution in Cu/MCM-22. The well-resolved EPR spectrum of Cu/MCM-22 powder displayed the characteristics of monomeric and axial Cu(II) sites (Fig. S25 and Table S15), with resolution of the Cu hyperfine interaction [50,51] (nuclear spin *I* = 3/2, combined 100% natural abundance). In CH_3_CN, the Cu^2+^ signal remained unchanged (Fig. 3B and Fig. S26), reflecting unpaired electrons in Cu^2+^ cations. Adding 1-undecene had minimal effect, but benzaldehyde, even under anaerobic conditions (glovebox-prepared samples), significantly reduced the Cu^2+^ signal, indicating reduction of Cu^2+^ to Cu^+^, which lacks unpaired electrons. O_2_ exposure restored the Cu^2+^ signal, suggesting oxidation of Cu^+^ back to Cu^2+^. A plausible pathway involves the coordination of O_2_ with the Cu^+^ center, forming a transient copper–oxygen adduct [e.g. a Cu(III)–oxo intermediate]. This activated oxygen species can then participate in the regeneration of the active Cu^2+^ site, potentially via hydrogen atom transfer from the reaction medium, with water as a likely byproduct [12,17]. This transformation likely involves benzaldehyde activation by Cu^2+^ via homolytic cleavage, yielding acyl and hydrogen radicals. The hydrogen radicals react with Cu–OH groups [evidenced by the 3728 cm^−1^ infra-red (IR) peak], forming H_2_O and reducing Cu^2+^ to Cu^+^.

Benzaldehyde adsorption and evolution on Cu/MCM-22 were studied using FTIR spectroscopy. Catalyst pellets were activated at 400°C under vacuum for 1 h to remove adsorbed water, cooled to 30°C, and a background spectrum was recorded. Benzaldehyde vapor introduced via a stainless-steel tube accumulated over time, leading to progressively stronger IR signals until saturation. The adsorption spectra revealed peaks at 1598, 1583, 1457 and 1313 cm^−1^ (benzene ring vibrations), 1706 cm^−1^ (bulk benzaldehyde carbonyl) and a 1695 cm^−1^ shoulder (adsorbed benzaldehyde carbonyl) [52]. Upon saturation of benzaldehyde adsorption, the Cu-loaded catalysts exhibited markedly stronger peak intensities than the Cu-free samples (Fig. S27A), indicating that Cu sites act as the primary adsorption centers. Following a 30 min of evacuation to remove physisorbed benzaldehyde, the evolution of adsorbed species was monitored by stepwise heating. With increasing temperature, even weakly chemisorbed benzaldehyde was gradually desorbed. At 200°C, the adsorption peaks on the Cu-free catalysts nearly disappeared, whereas Cu/MCM-22 retained much stronger adsorption signals compared to Cu/Al_2_O_3_ and Cu/SiO_2_, demonstrating that Cu sites in Cu/MCM-22 more effectively promote benzaldehyde adsorption (Fig. S27B). In addition, the bands at 1640 and 1670 cm^−1^, which were characteristic of water, diminished during gradual heating to 100 and 200°C, indicating H_2_O desorption from Cu/MCM-22 [53,54] (Fig. 3C). Given the dehydration pretreatment, H_2_O was likely generated *in situ* from benzaldehyde–Cu/MCM-22 interactions. An *in situ* FTIR study confirmed this; after activation at 400°C, benzaldehyde diffusion produced initial peaks at 1706, 1695, 1598, 1583, 1457 and 1313 cm^−1^. Over time, a broad 1600–1700 cm^−1^ band emerged, with filled valleys at 1640 and 1670 cm^−1^, indicating H_2_O formation (Fig. 3D). In contrast, the control experiment using SiO_2_ showed persistent 1640 and 1670 cm^−1^ valleys (Fig. S28), proving that H_2_O arose from Cu/MCM-22–benzaldehyde interactions, consistent with EPR and temperature-dependent FTIR results. Meanwhile, the characteristic Cu–OH peak decreased with increasing benzaldehyde adsorption (Fig. S29), indicating that the disappearance of Cu–OH was accompanied by the formation of H_2_O. This supports a mechanism where Cu–OH facilitates the cleavage of the C–H bond in benzaldehyde, leading to H_2_O generation. The dual role of Cu in adsorption and activation enhances epoxidation efficiency, though quantifying the contribution of this step to acylperoxy radical formation remains challenging.

### DFT results

Experimental findings revealed that isolated Cu–OH sites within MCM-22 react with benzaldehyde to generate acyl radicals, which transform into acylperoxy radicals, initiating epoxidation. To elucidate the underlying mechanisms, DFT calculations were employed to probe the formation and evolution of aldehyde-derived radicals at Cu–OH sites. 1-Butene was chosen as a model substrate to represent LAOs. The Cu/MCM-22 model, with Cu–OH anchored at Brønsted acid sites induced by framework aluminum, is depicted in Fig. S30A. For comparison, a model featuring tri-coordinated Al sites in MCM-22 was constructed to assess benzaldehyde activation (Fig. S30B).

**Figure 4.**
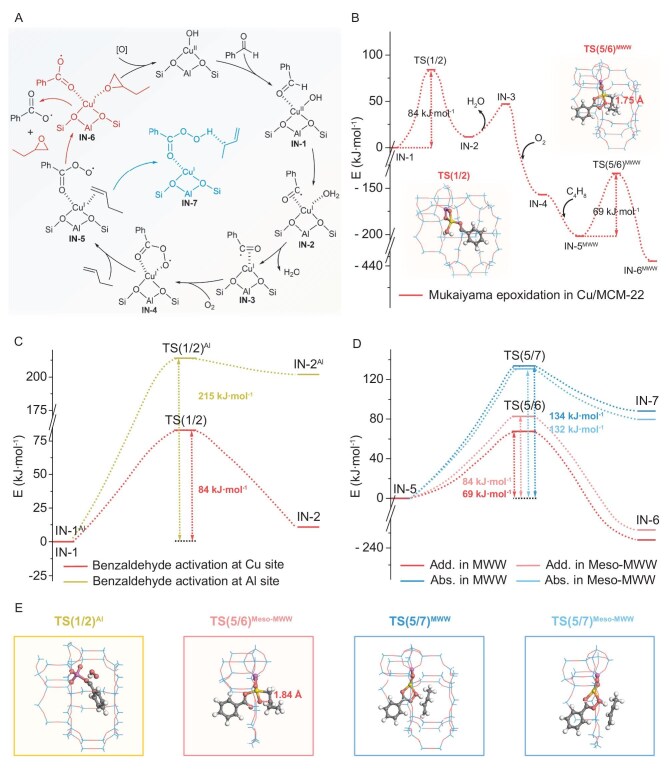
The proposed mechanism and theoretical study of Mukaiyama epoxidation on Cu/MCM-22. (A) The reaction mechanism based on experimental results. The desired and undesired pathways are shown in red and blue, respectively. (B) Electron energy profile and corresponding optimized transition states for 1-butene epoxidation on Cu/MCM-22. TS(1/2) and TS(5/6)^MWW^ denote the transition states of benzaldehyde activation at the Cu site and addition pathway in MWW. (C and D) Electron energy profiles for benzaldehyde activation at the Cu site and the Al site (C); addition and abstraction pathways on the MWW and Meso-MWW models (D). (E) Transition states for key steps: TS(1/2)^Al^, TS(5/6)^Meso-MWW^, TS(5/7)^MWW^ and TS(5/7)^Meso-MWW^. The Cu, Al, Si, O, C and H atoms are shown in yellow, purple, blue, red, gray and white, respectively.

The proposed mechanism for Cu/MCM-22-catalyzed Mukaiyama epoxidation, with benzaldehyde as the sacrificial reductant, is illustrated in Fig. 4A. Benzaldehyde adsorbs onto the Cu–OH site (IN-1), where the Cu–OH hydroxyl group abstracts a hydrogen from the aldehyde, forming H_2_O and an acyl radical (IN-2), accompanied by a Cu oxidation state change. Following H_2_O desorption (IN-3), the acyl radical captures O_2_ to form an acylperoxy radical (IN-4). Within the confined pores of MCM-22, an olefin was adsorbed on Cu (IN-5) and then reacted with an acylperoxy radical to yield the epoxide (IN-6) with minimal allyl byproducts (IN-7). The electronic energy profile and structures of 1-butene epoxidation intermediates are shown in Fig. 4B and Fig. S31. The energy profile identifies benzaldehyde activation (IN-1 to IN-2) as the rate-limiting step, with a barrier of 84 kJ mol^−1^, underscoring its critical role in epoxidation. Subsequent steps (IN-2 to IN-5) involve low-barrier adsorption and desorption processes.

The role of Cu–OH in benzaldehyde activation was compared to tri-coordinated Al sites, which leverage Lewis acidity [28]. As shown in Fig. S32, benzaldehyde adsorption on Cu–OH forms an acyl radical and H_2_O, whereas Al sites, reacting with O_2_, yield an acyl radical and an HOO· radical. The energy barrier for benzaldehyde activation at Cu–OH is 84 kJ mol^−1^, 131 kJ mol^−1^ lower than at Al sites (215 kJ mol^−1^, Fig. 4C), highlighting the superior efficacy of Cu–OH in generating acyl radicals. Cu(II) accepts an electron from the coordinated aldehyde, promoting proton loss, C–H bond cleavage and acyl radical formation. Intramolecular dehydration of H radicals with hydroxyl groups forms H_2_O, further facilitating this step.

As depicted in Fig. 4A, the acylperoxy radical in IN-5 reacts with the C=C bond to form epoxides or with allyl hydrogens to produce byproducts. The high epoxide selectivity of Cu/MCM-22 prompted modeling of Meso-MWW, lacking confined channels (Fig. S33), to compare with MWW (MCM-22). Energy profiles for addition (epoxide formation) and abstraction (byproduct formation) pathways revealed that the addition barrier in MWW (69 kJ mol^−1^) is lower than in Meso-MWW (84 kJ mol^−1^, Fig. 4D), indicating that the confined zeolite channels in MCM-22 facilitate the addition reaction by reducing the activation energy required for epoxide formation. Conversely, the abstraction barrier in MWW is 134 kJ mol^−1^, slightly higher than in Meso-MWW. Further evidence was provided by the transition state analysis of addition pathways. Compared with Meso-MWW, the confined space of MWW brought the acylperoxy radical and olefin into closer proximity (1.75 Å compared to 1.84 Å in Meso-MWW), promoting the formation of epoxide (Fig. 4B and E, Figs S34 and S35).

In summary, DFT calculations reveal that Cu–OH in Cu/MCM-22 significantly lowers the benzaldehyde activation barrier, enhancing reaction activity. The confined channels of MCM-22 impose critical constraints on intermediates like acylperoxy radicals, ensuring exceptional chemoselectivity. These insights illuminate the role of Cu-based zeolite catalysts in epoxidation and guide the rational design of advanced catalysts for optimized performance.

## CONCLUSIONS

This study presents the development and mechanistic exploration of a Cu/MCM-22 catalyst for the highly selective Mukaiyama epoxidation of linear LAOs. Through integrated experimental and theoretical analyses, we established that Cu–OH active sites within the confined microenvironment of the MCM-22 zeolite significantly enhance catalytic performance, chemoselectivity and aldehyde coupling efficiency. Advanced characterization confirmed that Cu^2+^ ions, atomically dispersed within the MCM-22 framework, efficiently activate benzaldehyde to form acyl radicals, accompanied by H_2_O production and Cu oxidation state changes. These radicals, stabilized by the zeolite’s confined pores, are steered away from undesired pathways, such as hydrogen atom abstraction and radical recombination, toward the preferred radical addition route. DFT calculations corroborated the kinetic and thermodynamic advantages of Cu–OH sites, revealing reduced energy barriers for benzaldehyde activation (84 kJ mol^−1^) and the addition pathway, alongside increased steric hindrance for abstraction within the zeolite channels. Catalytic evaluations demonstrated the exceptional performance of Cu/MCM-22, achieving 97% conversion and 90% selectivity for 1-undecene epoxidation, with aldehyde coupling efficiency rising from 25% to 60%. In contrast, Cu/SiO_2_ and Cu/Al_2_O_3_ catalysts, lacking confinement, exhibited lower selectivity due to competing side reactions.

These findings underscore the potential of integrating tailored metal active sites within zeolite confinement to enhance efficiency and selectivity in radical-driven catalysis. Cu/MCM-22 not only offers practical utility for epoxide synthesis but also serves as a platform for designing advanced catalysts with optimized active sites and structural environments for complex transformations. Future efforts could explore refining zeolite compositions for broader substrate compatibility and enhancing long-term stability under industrial conditions. This work advances the understanding of confinement effects in catalysis, illuminating a path toward sustainable, selective chemical processes.

## Supplementary Material

nwaf502_Supplemental_File
