# Supplementary material for: Synergistic Cu–OH and zeolite confinement in Cu/MCM-22 for benzaldehyde-mediated Mukaiyama epoxidation of long-chain α-olefins
Source: Natl Sci Rev. 2025 Nov 14;13(3):nwaf502. doi: 10.1093/nsr/nwaf502 (PMC12866670; doi:10.1093/nsr/nwaf502)
Supplement: nwaf502_Supplemental_File [file nwaf502_supplemental_file.pdf]

Supplementary Information for

Synergistic Cu-OH and zeolite confinement in Cu/MCM-22 for benzaldehyde-mediated Mukaiyama epoxidation of long-chain α-olefins

Hongying Chang[1,3,#], Ziyu Zhou[1,2,3,#], Peng He[2,3]*, Kun Zhang[1,2,3], Yujie Xie[1,2,3], Xiangjie Zhang[1,2,3], Tao Yan[1,2,3], Min Zhang[1,2,3], Gaolei Qin[1,2,3], Huaming Hou[3], Yanyan Chen[2], Jianguo Wang[1]*, Zhi Cao[2,3]*

[1]School of Chemical Sciences, University of Chinese Academy of Sciences, Beijing 100190, P. R. China;

[2]State Key Laboratory of Coal Conversion, Institute of Coal Chemistry, Chinese Academy of Sciences, Taiyuan 030001, P. R. China;

[3]National Energy Center for Coal to Clean Fuels, Synfuels China Technology Co., Ltd., Beijing 101407, P. R. China

***Corresponding author.**

E-mail: hepeng@sxicc.ac.cn; caozhi@sxicc.ac.cn; wangjianguo@ucas.ac.cn

[#]Equally contributed to this work.

This PDF file includes:

Supplementary Text

Scheme S1

Figures S1 to S35

Tables S1 to S15

SI References

Supplementary Text

Materials

1-undecene ($C_{11}H_{22}$, 99%, TCI), benzaldehyde (C_7H_6O, 99%, Aladdin), acetonitrile (CH_3CN, 96%, Fisher), n-nonane (C_9H_{20}, 99%, Aladdin), *N-tert*-butyl-α-phenylnitrone (PBN, 98%, Innochem), copper nitrate hydrate ($Cu(NO_3)_2 \cdot 3H_2O$, AR, Macklin), 2,6-ditert-butyl-4-methylphenol (BHT, 99%, Innochem), Triphenylamine (TPA, 98%, Innochem), 2-Phenylquinoline (PQ, 99%, Innochem), ethanol (C_2H_6O, 99%, Alfa), 1-heptene (C_7H_{14}, 98%, Aladdin), 1-octene (C_8H_{16}, 99%, Aladdin), 1-nonene (C_9H_{18}, 95%, Aladdin), 1-decene ($C_{10}H_{20}$, 96%, Alfa), MCM-22 zeolite (Na-form, Si/Al=12.5, Adamas). All chemicals were used without further purification.

Preparation of catalysts

The Cu/MCM-22 catalyst was prepared via ion exchange of Na-type MCM-22 (Si/Al = 12.5). $Cu(NO_3)_2 \cdot 3H_2O$ was dissolved in deionized water to yield an aqueous solution with a pH of 4.5-5.5. The MCM-22 zeolite was immersed in this solution at 40 ℃ with continuous stirring for 12 hours. Following ion exchange, the mixture was cooled to ambient temperature, and unexchanged Cu^{2+} ions were removed by centrifugation and three consecutive washes with deionized water. The resulting solid was dried at 80 ℃ in air for 12 hours and calcined in static air at 550 ℃. Cu/Al_2O_3 and Cu/SiO_2 catalysts were synthesized using an analogous procedure.

Preparation of modifier-Cu/MCM-22 catalysts

The Cu/MCM-22 catalyst was immersed in ethanol containing excess poisoning agents (triphenylamine or 2-phenylquinoline). The mixture was ultrasonically dispersed for 3 minutes and stirred at 50 ℃ for 2 hours. The modifier-Cu/MCM-22 catalyst was then separated from the solution by centrifugation. Ethanol was evaporated at 80 ℃, and the sample was subsequently dried at 110 ℃.

Characterizations

The elemental composition of the samples was determined by inductively coupled plasma-optical emission spectroscopy (ICP-OES) using an Optima 2100DV spectrometer (PerkinElmer). The X-ray diffraction (XRD) patterns were recorded on a Bruker D8 Advance diffractometer with a step size of 0.02°, a scan rate of 0.2 ° s^{-1}, and a scan range of 5 ° to 80 ° using a Cu Kα radiation source (40 kV, 40 mA). Surface area data of these samples were determined from N_2 adsorption-desorption isotherms at -196 ℃ on the Micromeritics ASAP 2020 analyzer and ASAP 2420 analyzer. Prior to analysis, the samples were first evacuated at 300 ℃ for 5 hours. Total surface area was calculated using the Brunauer-Emmett-Teller equation. The microporous area, external surface area, and micropore volume were determined using the t-plot method. The pore size was calculated using the Barrett-Joyner-Halenda (BJH) analysis and the microporous size was determined using the Horvath-Kawazoe (HK) method.

The morphology of all samples was observed by an FEI Quanta 400F scanning electron microscope (SEM) with an electron acceleration voltage of 10 kV. HAADF-STEM images and EDX mapping were taken with Talos™ F200A at 200 kV acceleration voltage. The ADF-STEM images were collected at 300 kV with aberration-corrected STEM (FEI, Titan Cubed Thermis G2 and JEOL, JEM-ARM300F). For all data, either 2048 × 2048-pixel or 1024 × 1024-pixel images were recorded and the dwell time for each pixel was varied from 3 μs up to 12 μs. The beam current used was below 10 pA.

X-ray photoelectron spectroscopy (XPS) measurements were performed on a Thermo Scientific K-Alpha system equipped with a monochromic Al Kα X-ray source. All binding energies were referenced to the C 1s peak at 284.8 eV of the surface adventitious carbon to correct the shift caused by charge effect.

The XAS study at the Cu K-edge was carried out at the BL14W1 and BL13SSW beamlines

of the Shanghai Synchrotron Radiation Facility, using a Si(311) double-crystal monochromator. Data were recorded in fluorescence yield mode, using a four-element Ge detector. Energy calibration was achieved using Cu foil and the tabulated edge energy was defined as the first inflection point in the derivative spectrum. ATHENA and ARTEMIS software were used for data collation and analysis. The data were fitted in R-space with theoretical models constructed from FEFF based on the crystal structures of Cu metal and DFT-optimized Cu/MCM-22.

FT-IR measurements were collected using the Bruker VERTEX 70v spectrometer equipped with a mercury cadmium telluride detector. The spectra were recorded in transmission mode, in the range of 1000-4000 cm^{-1}, with an average of 64 scans and a resolution of 3 cm^{-1}. Approximately 30 mg samples were pressed into self-supporting wafers with a diameter of 13 mm and loaded into a specially designed high-temperature infrared chamber equipped with a BaF_2 window and connected to a vacuum adsorption unit that allows dynamic pressure control. Before each sample introduction, the empty chamber was baked at 500 ℃ for 2 hours to ensure the complete removal of organic residues.

(i). When the experiment to measure hydroxyl was performed, the spectrum of the cavity sample pool was used as the background. After the sample was put in, the temperature rate was increased to 400 ℃ at 10 ℃ min^{-1}, and the sample was calcined for 1 hour. The infrared spectrum of the hydroxyl was measured when the sample was cooled.

(ii). Before the experiment, benzaldehyde was frozen in bottles using liquid nitrogen, evacuated for over five minutes, and then thawed. This process was repeated three times to remove any dissolved water and oxygen from the solvent. When the adsorption experiment of benzaldehyde was conducted, the catalyst was pretreated before measurements. The samples were degassed at 400 ℃ in vacuum for 1 hour. The spectral data of the pretreated catalyst was used as the background. Benzaldehyde was adsorbed onto the catalyst at room temperature of 30 ℃ until saturation. Then He was blown away for 30 minutes to remove the physically adsorbed benzaldehyde. The catalyst was heated at a rate of 10 ℃ min^{-1} to 50 ℃, 100 ℃, and 200 ℃, and kept at each temperature for 30 minutes. After being cooled to room temperature, it is time to measure the corresponding temperature of benzaldehyde desorption curve.

EPR spin-trapping test: 5.0 mL of acetonitrile, 5 mmol of benzaldehyde, and 20 mg of catalyst were added to a 25 mL Schlenk tube. Then the reactants were placed in a constant temperature at 60 ℃ and stirred at 600 rpm under anaerobic and aerobic conditions for a certain period. The EPR spin trap experiments employed *N-tert*-butyl-α-phenylnitrone (PBN) as a spin-trapping agent to analyze the radicals in the reaction mixture. Typically, 10 mg PBN was added to 500 μL reaction mixture to form a spin-trapping mixture. An aliquot of the mixture was shifted into a 50 μL tubing capillary tube (intra MARK), which was then placed in a quartz X-band EPR tube. For quantitative comparison of the EPR signal intensities, the same amount of suspension in the capillary was used for the test. The EPR spectra were recorded at room temperature on a Bruker EMX-Plus Spectrometer. Relevant parameters are as follows: a center field of 3517.3 G, a sweep time of 30 s, and a microwave power of 20 dB.

Operando EPR tests: In a nitrogen atmosphere glovebox, 5.0 mL of acetonitrile and 20 mg of catalyst were added to a 25 mL Schlenk tube as a blank control group. In the experimental group, 1-undecene, benzaldehyde, 1-undecene and benzaldehyde were added to the corresponding tubes to control for a single experimental variable. Then the reactants were placed in a constant temperature at 60 ℃ and stirred at 600 rpm in a N_2 atmosphere for a certain period. An aliquot of that solution was transferred by micropipette to a dry X-band EPR tube, and removed from the glovebox. The sample was degassed by three freeze pump-thaw cycles, then flame sealed under vacuum. The sample was frozen in dry ice and was kept frozen until and throughout the EPR measurement. The operando EPR spectra were recorded on a Bruker EMX-Plus Spectrometer under 100 K. A modulation amplitude, modulation frequency and

microwave power of 4 G, 9.4347 GHz and 2 mW were used, respectively. Theoretical modelling of EPR spectra was performed with EasySpin package (Version 6.0.0) embedded in MATLAB version R2020a[1].

Catalytic performance evaluation

Prior to reactions, acetonitrile was saturated with O_2 by bubbling for 30 minutes to enhance mass transfer. In a typical experiment, a 25 mL Schlenk tube was charged with 5.0 mL of acetonitrile, 1.0 mmol of olefin, 5.0 mmol of benzaldehyde, and 20 mg of catalyst (corresponding to 0.157 mol% Cu). The mixture was maintained at 60 ℃ and stirred at 600 rpm under an O_2 atmosphere for 6 hours. When air was employed as the oxidant, the O_2 was replaced by air, with all other experimental parameters kept identical. After cooling to ambient temperature, 50.0 μL of n-nonane was added as an internal standard. The catalyst was separated by centrifugation, and the supernatant was filtered for analysis using a pre-calibrated Agilent 7890B gas chromatograph equipped with a flame ionization detector (FID) and an HP-5 column.

The apparent activation energy (E_a) for epoxidation of 1-undecene was estimated within the temperatures range from 40 ℃ to 70 ℃. These measurements were taken over a short reaction time of 20 minutes according to different temperatures, to maintain the conversions lower than 20%. The calculation formula is as follows:

$$lnr = -\frac{E_a}{RT} + lnA$$

where r, E_a, and A represent the reaction rate (mol g^{-1} s^{-1}), apparent activation energy (kJ mol^{-1}), and pre-exponential factor, respectively.

The carbon balance calculation equation is as follows:

$$\text{Carbon balance}(\%) = \frac{C_{\text{unconverted olefin}} + C_{\text{generated epoxide}} + C_{\text{generated allylic byproducts}}}{C_{\text{feed of olefin}}} \times 100\%$$

where $C_{\text{unconverted olefin}}$ is the moles of unconverted olefin in the mixture after the reaction, $C_{\text{generated epoxide}}$ is the moles of epoxide produced during the reaction, $C_{\text{generated allylic byproducts}}$ is the moles of allylic byproducts produced during the reaction, and $C_{\text{feed of olefin}}$ is the moles of the olefin feedstock in the reaction matrix.

Computational details

The framework of MCM-22 zeolite (denoted as MWW) was simulated with 49T cluster model in terms of the silicate analogue structure in the database of the Structure Commission of the International Zeolite Association (IZA). The Cu/MCM-22 model was constructed by locating the Cu atom at the junction of the 10MR and 12MR rings of the MCM-22 zeolite. In this model, the isolated Cu-OH species was anchored at Brønsted acid sites induced by the four-coordinated Al species. To investigate the role of Cu-OH sites in the activation of benzaldehyde, a theoretical model of Al-MCM-22 with a tri-coordinated aluminum site in MCM-22 was also constructed for comparison (Figure S30). Additionally, to explore the confinement effect of the MCM-22 channels in controlling the formation of intermediates, a theoretical model of Meso-MWW with 25T cluster was constructed in a similar manner (Figure S33).

The computational study was performed using the Gaussian 16 package. All geometry optimizations and frequency calculations were carried out using the B3LYP functional with the 6-31G(d,p) basis set. Single-point energy calculations were performed using the 6-311++G(2df,2p) basis set to ensure high accuracy[2].

To study the epoxidation reaction of benzaldehyde and 1-butene on the Cu/MCM-22 zeolite, the adsorption structures of the reactants, intermediates, and products were optimized to local minima, and the transition states (TSs) were optimized to the saddle point. The local

minima were confirmed by the absence of imaginary frequencies, while the transition states were verified by the presence of a single imaginary frequency. The quasi-internal reaction coordinate (quasi-IRC) approach was used to ensure that each transition state correctly connects the corresponding reactants and products.

The adsorption energy of the reactants (benzaldehyde, O_2 and 1-butene) on the Cu/MCM-22 zeolite was calculated using the following formula:

E_{ads}(1-butene) = E(1-butene@zeolite) - [E(1-butene) + E(zeolite)]

where E(1-butene@zeolite), E(1-butene), and E(zeolite) are the energies of the zeolite with the adsorbed reactant, the free reactant, and the zeolite alone, respectively.

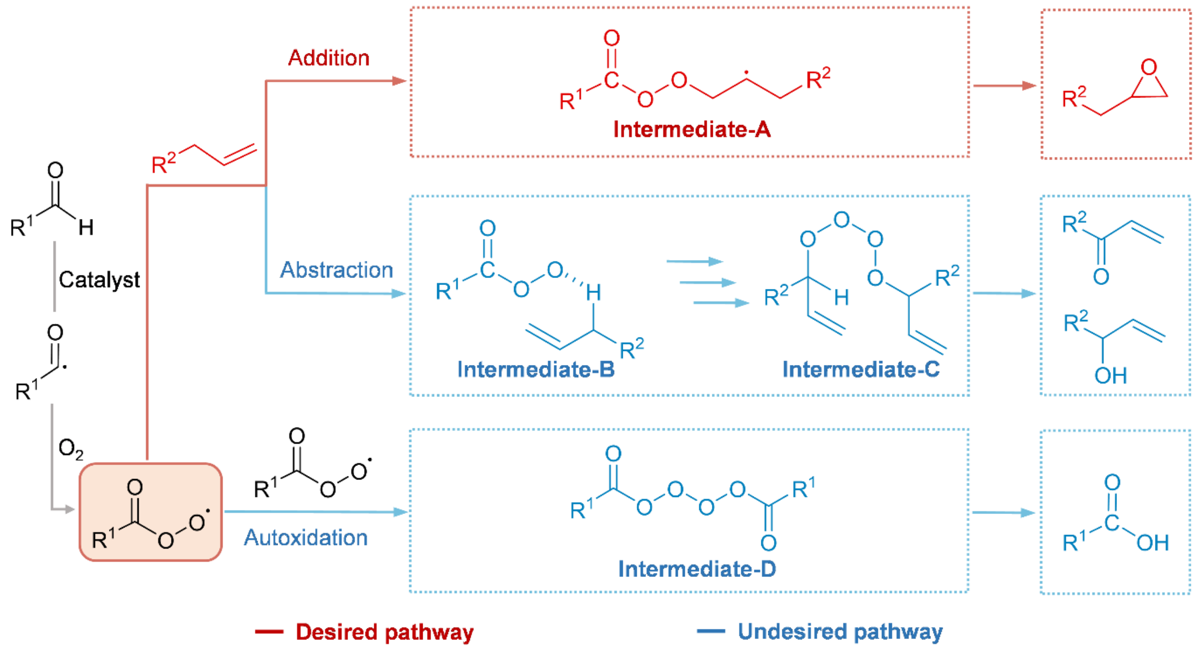

Scheme S1. Reaction pathways of Mukaiyama epoxidation.

In the radical chain mechanism, aldehydes are activated by catalysts to produce acyl radicals, thereby initiating a radical reaction. The acyl radical and sufficient oxygen rapidly generate acylperoxy radicals, which act as primary oxidizing species that adduct with olefins to form epoxidation products (addition pathway). At the same time the acylperoxy radical can also react with the olefin through hydrogen atom abstraction (HAA) pathway to form allylic oxidation byproducts. Furthermore, acylperoxy radicals can undergo self-reactions (autoxidation pathway) resulting in suboptimal aldehyde coupling efficiency.

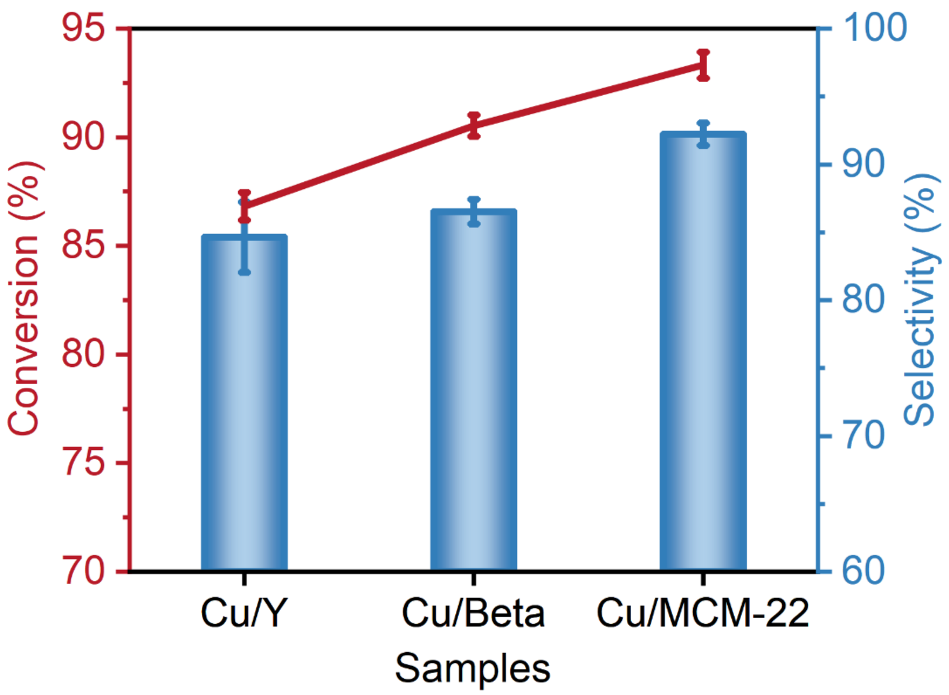

Figure S1. Catalytic performance of Mukaiyama epoxidation of 1-undecene catalyzed by Cu/MCM-22, Cu/Beta and Cu/Y samples.

The error bars of conversion of 1-undecene and selectivity for 1,2-epoxyundecane are calculated based on three independent measurements. Reaction conditions: 5.0 mL of acetonitrile, 1.0 mmol of 1-undecene, 5.0 mmol of benzaldehyde, O_2 balloon, 20.0 mg of catalyst, 6 hours, 600 rpm.

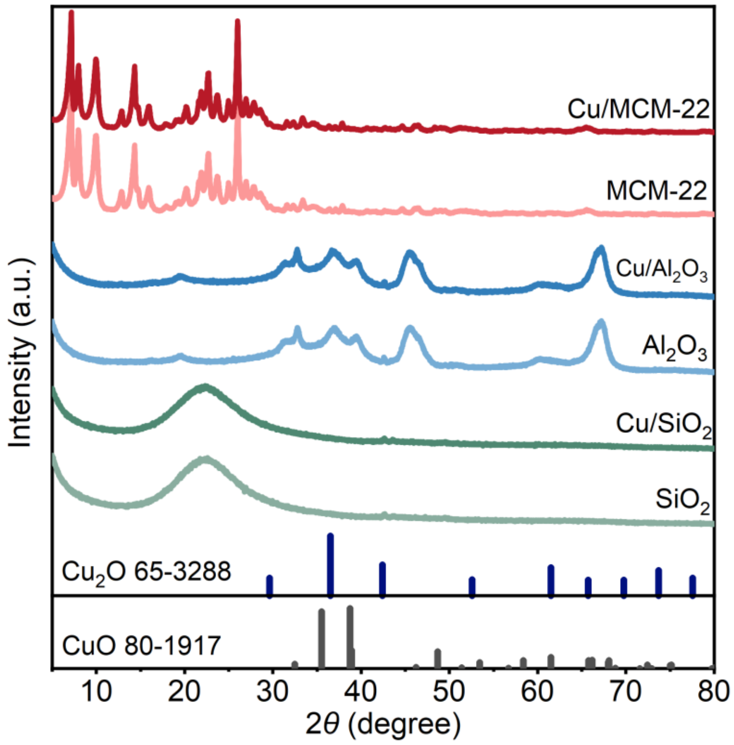

Figure S2. XRD patterns of Cu/MCM-22, MCM-22, Cu/Al_2O_3, Al_2O_3, Cu/SiO_2 and SiO_2 samples.

The XRD patterns of Cu/MCM-22, Cu/Al_2O_3 and Cu/SiO_2 revealed the absence of CuO_x peaks, suggesting that Cu was highly dispersed within the Cu/MCM-22.

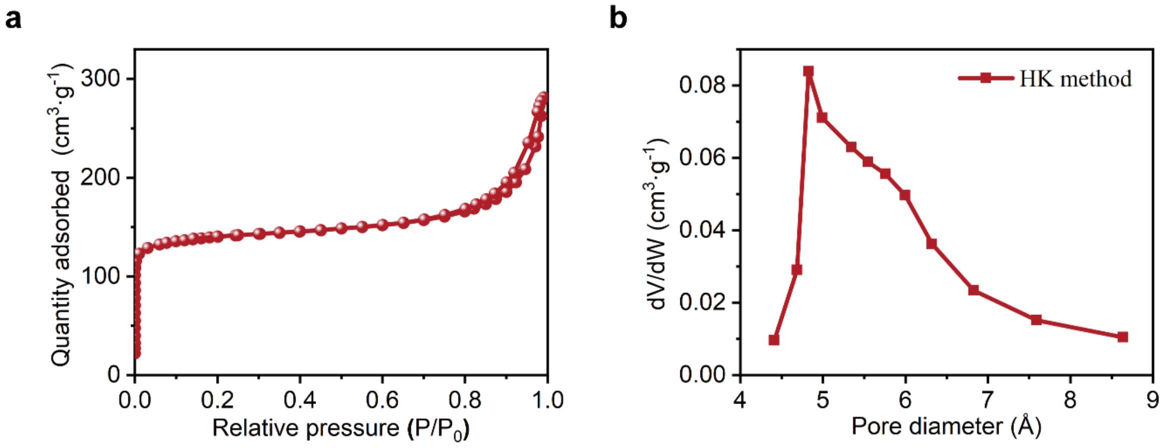

Figure S3. Textual and porosity properties characterized by N_2 sorption analysis of Cu/MCM-22 sample.

(a) N_2 physisorption isotherms of Cu/MCM-22, confirming the microporous structure of the zeolite. (b) The micropore size distribution curve of Cu/MCM-22 analyzed by HK model.

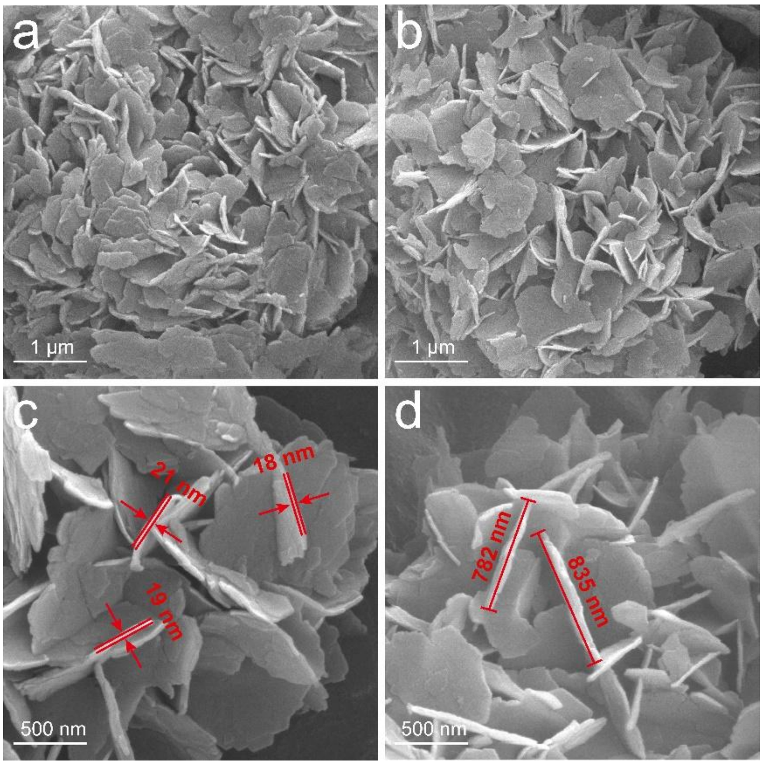

Figure S4. SEM images of Cu/MCM-22 sample.

A rose-like morphology with loosely aggregated disc-shaped particles was observed, showing the typical morphology of MWW zeolite. These disc-shaped particles measured about 800 nm and were made of thin slices with 15-25 nm thickness.

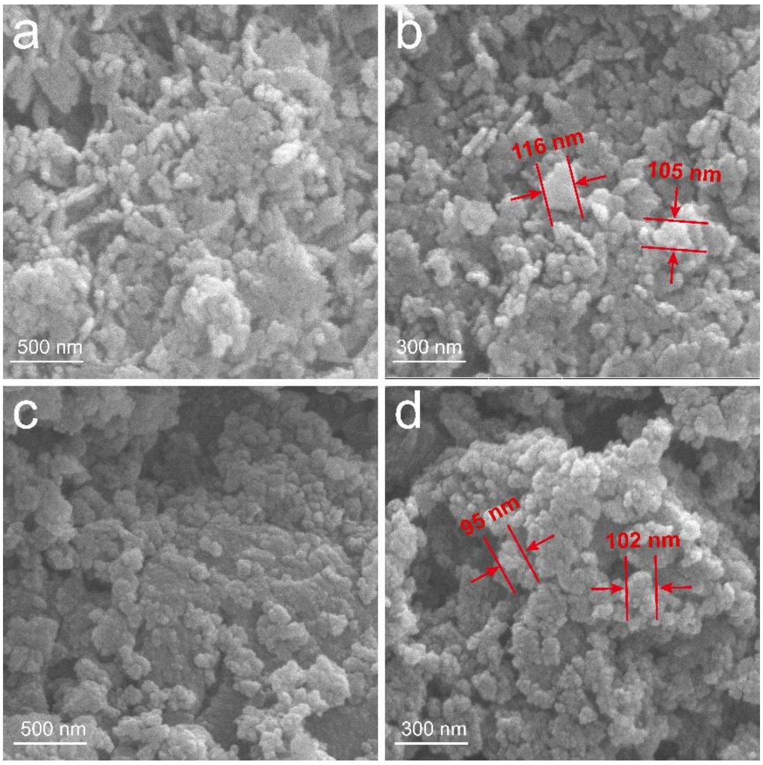

Figure S5. SEM images of (a,b) Cu/Al_2O_3 and (c,d) Cu/SiO_2.

The particle sizes of the Cu/SiO_2 and Cu/Al_2O_3 samples were estimated to be about 100 nm.

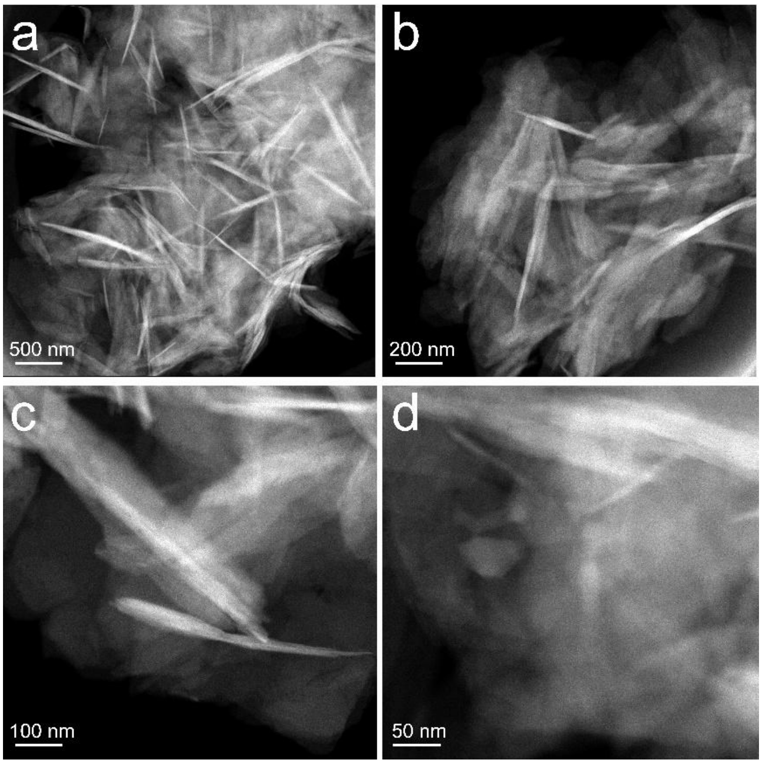

Figure S6. HAADF-STEM images of Cu/MCM-22 sample.

It was clear from the above images that no Cu clusters or particles were observed.

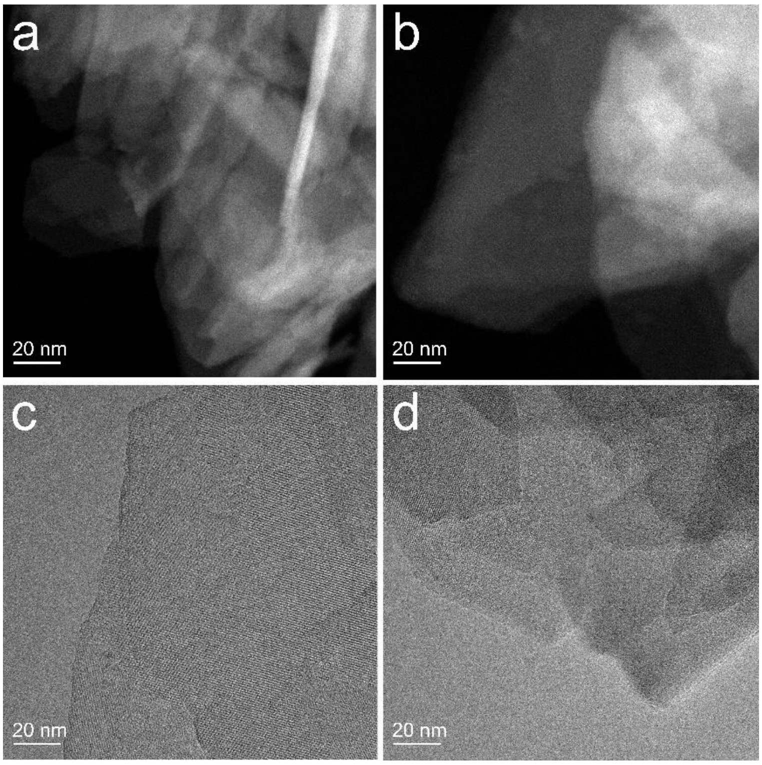

Figure S7. TEM images of Cu/MCM-22 sample.

The more detailed TEM images revealed the MCM-22 zeolite exhibited layer stacking, but there were still no Cu particles.

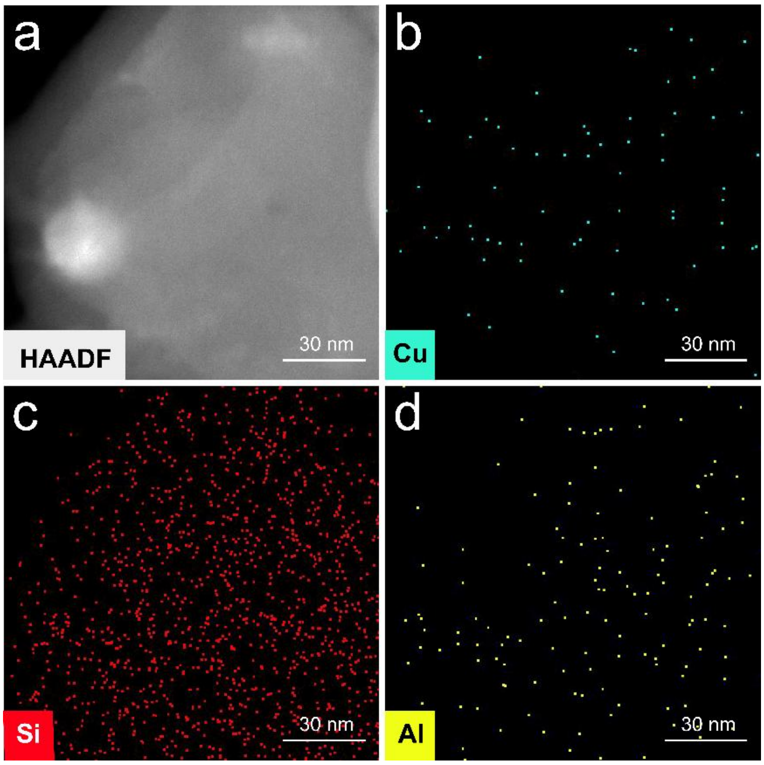

Figure S8. Representative HAADF-STEM and corresponding EDX mapping images of Cu/MCM-22 sample.

The light blue spot definitively confirmed a homogeneous distribution of Cu species over the MCM-22 zeolite crystallites. Corresponding EDS elemental mapping for copper (light blue), silicon (Red), aluminum (yellow), respectively.

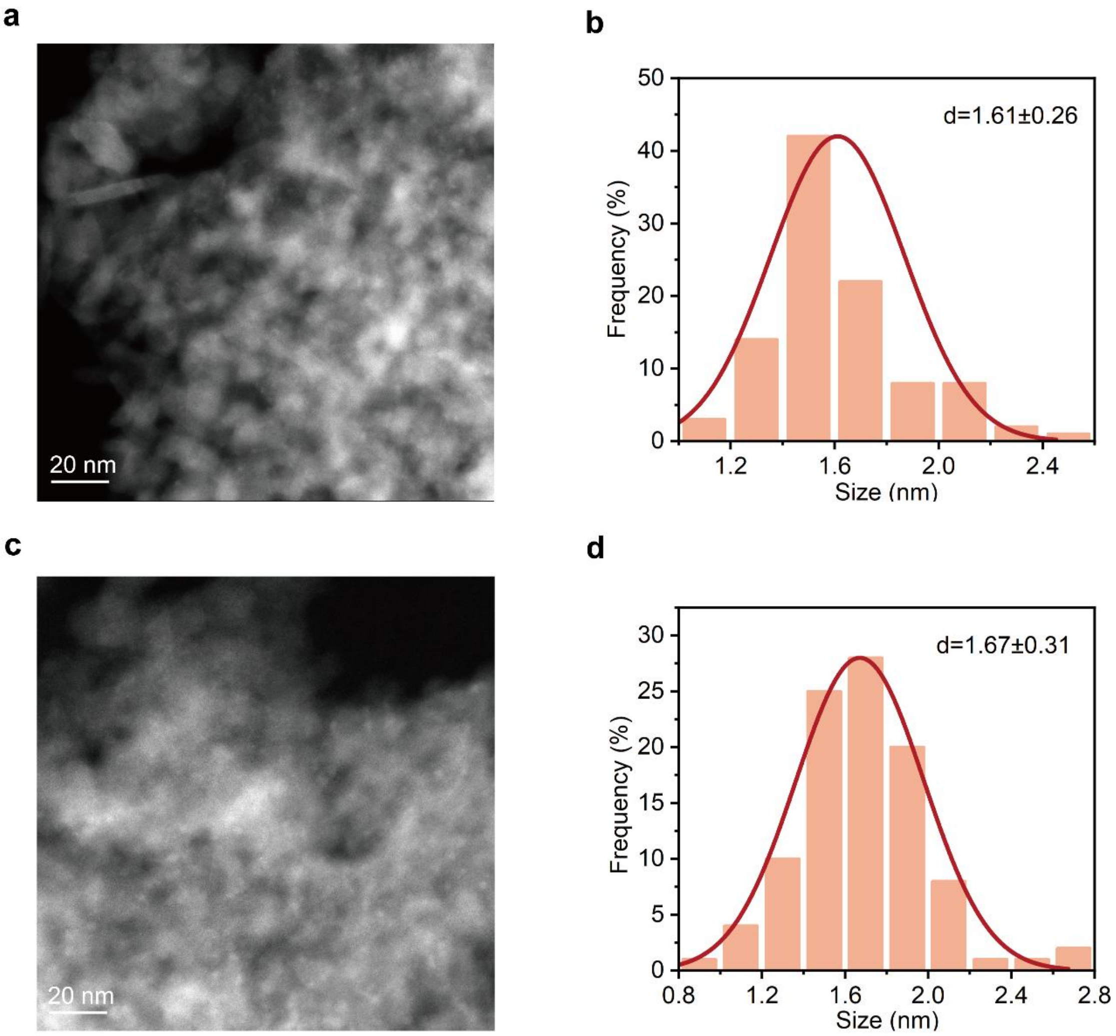

Figure S9. HAADF-STEM images and corresponding size distributions of metal particles for (a, b) Cu/Al_2O_3 as well as (c, d) Cu/SiO_2.

The presence of Cu/Al_2O_3 and Cu/SiO_2 was indicated by the observation of small particles with an average size of around 1.6 nm, suggesting the existence of CuO nanoparticles.

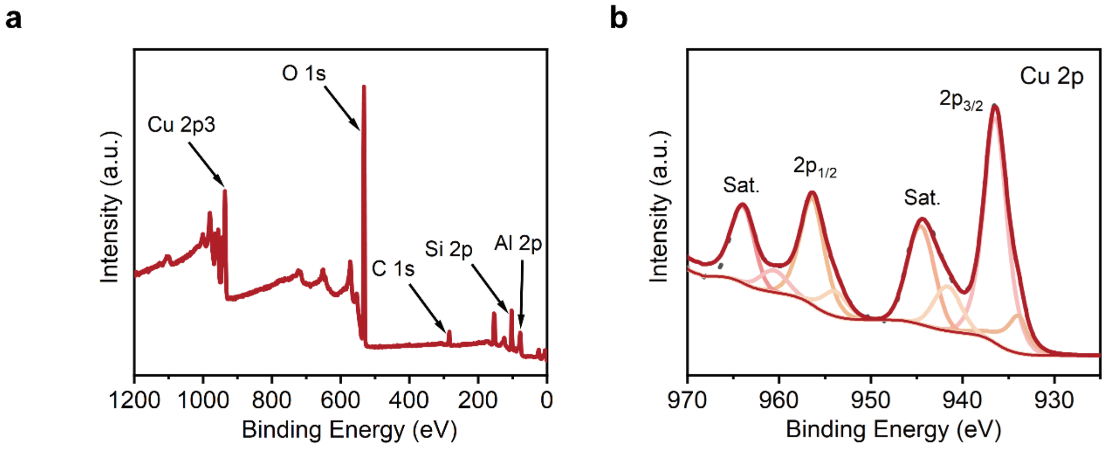

Figure S10. (a) XPS survey spectrum and (b) XPS high-resolution spectrum in the Cu 2p region of Cu/MCM-22 sample.

The observation of the Cu $2p_{3/2}$ and $2p_{1/2}$ binding energy peaks, in conjunction with their satellite peaks, served to substantiate the Cu(II) oxidation state.

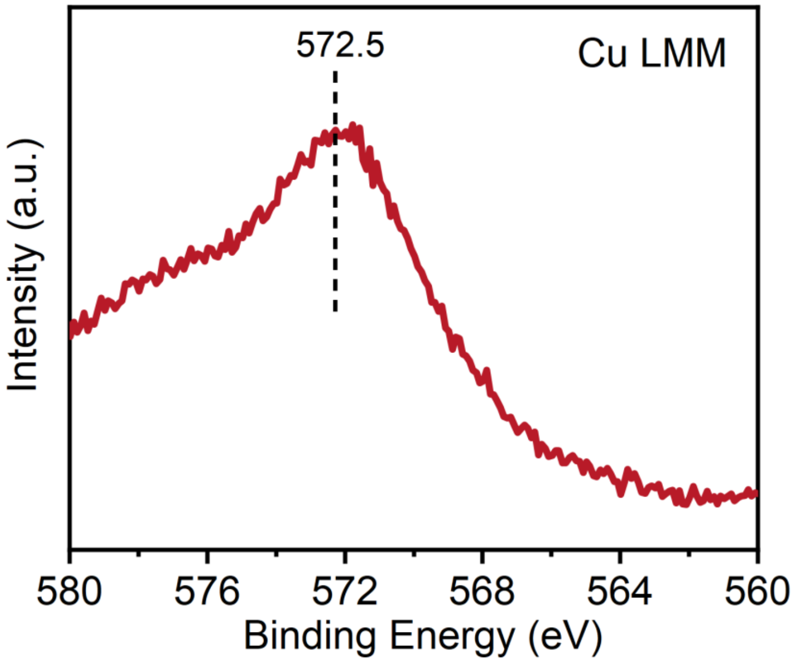

Figure S11. The Cu LMM XPS spectrum for Cu/MCM-22 sample.

The peak of 572.5 eV indicated that Cu existed in the form of Cu(II).

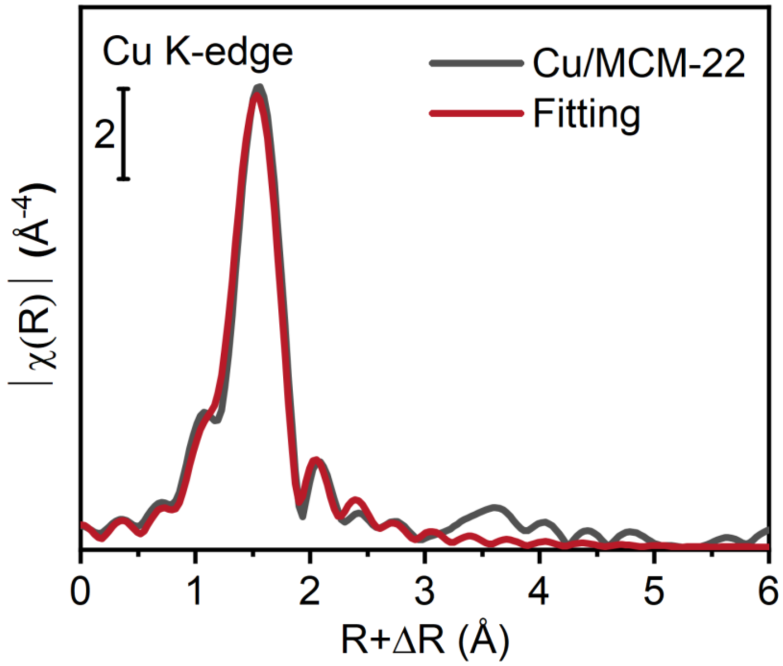

Figure S12. EXAFS fitting curve of Cu/MCM-22 sample.

The gray line represented the spectrum of the Cu/MCM-22 sample tested and the red represented the fitted spectrum.

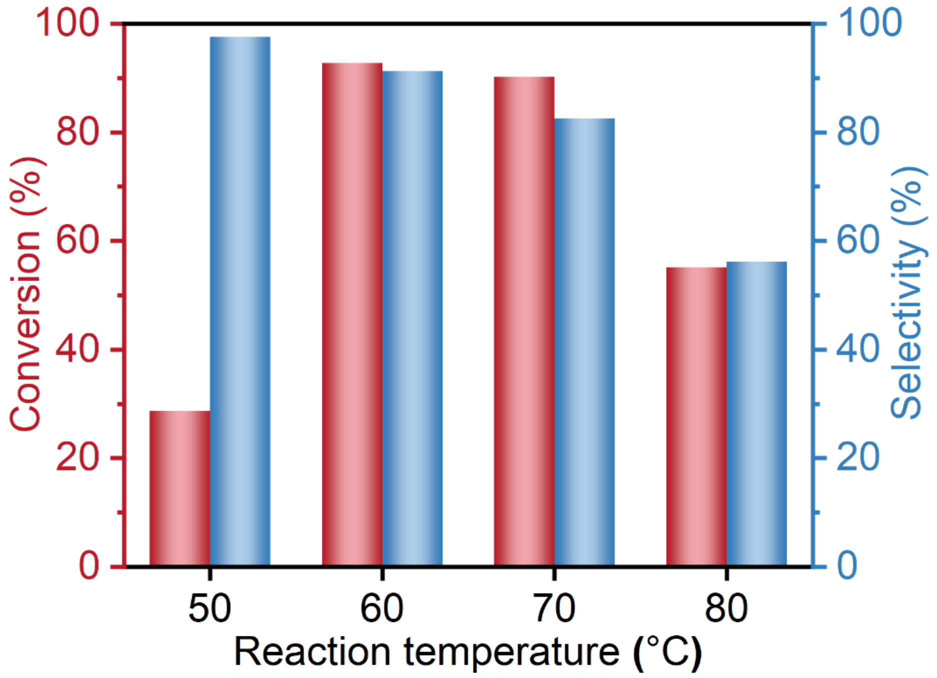

Figure S13. The optimization of the reaction temperature for the Mukaiyama epoxidation of 1-undecene catalyzed by Cu/MCM-22.

This figure illustrated the effect of reaction temperature on the conversion (red bars) and selectivity (blue bars) in the Mukaiyama epoxidation of 1-undecene. The results showed that as the temperature increased, the conversion rate initially rose before declining. At 50 ℃, the conversion was relatively low but increased significantly at 60 ℃, reaching its peak. Conversion remained high at both 60 ℃ and 70 ℃, then dropped slightly at 80 ℃. This result indicates that elevated temperatures facilitate the conversion of benzaldehyde to acyl radicals; however, when the formation rate of acyl radicals becomes excessively high, the resulting acylperoxy radicals undergo self-reactions, which in turn reduce the olefin conversion. Selectivity steadily decreased as the temperature increased. Based on these findings, 60 ℃ was identified as the optimal reaction temperature. Reaction conditions: 5.0 mL of acetonitrile, 1.0 mmol of 1-undecene, 5.0 mmol of benzaldehyde, O_2 balloon, 20.0 mg of catalyst, 6 hours, 600 rpm.

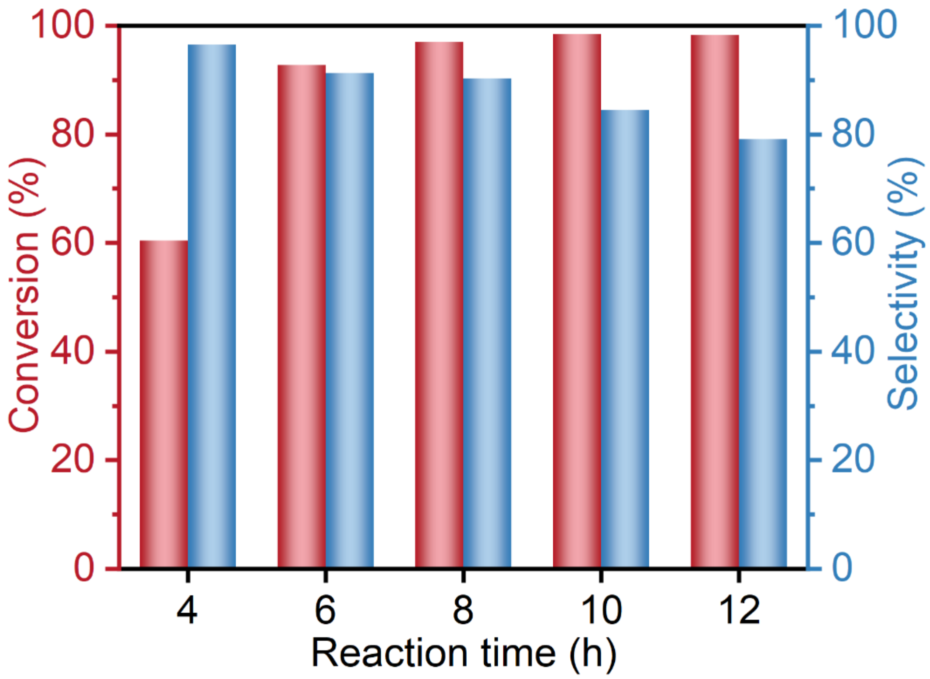

Figure S14. The optimization of the reaction time for the Mukaiyama epoxidation of 1-undecene catalyzed by Cu/MCM-22.

This figure illustrated the effect of reaction time on the conversion (red bars) and selectivity (blue bars) in the Mukaiyama epoxidation of 1-undecene. Reaction times were set at 4, 6, 8, 10, and 12 hours. The conversion of 1-undecene gradually increased with reaction time, reaching over 90% at 6 hours. However, as the reaction time was extended, by-products were produced sequentially, leading to a gradual decrease in selectivity. Based on these findings, 6 hours was identified as the optimal reaction time. Reaction conditions: 5.0 mL of acetonitrile, 1.0 mmol of 1-undecene, 5.0 mmol of benzaldehyde, O_2 balloon, 20.0 mg of catalyst, 60 ℃, 600 rpm.

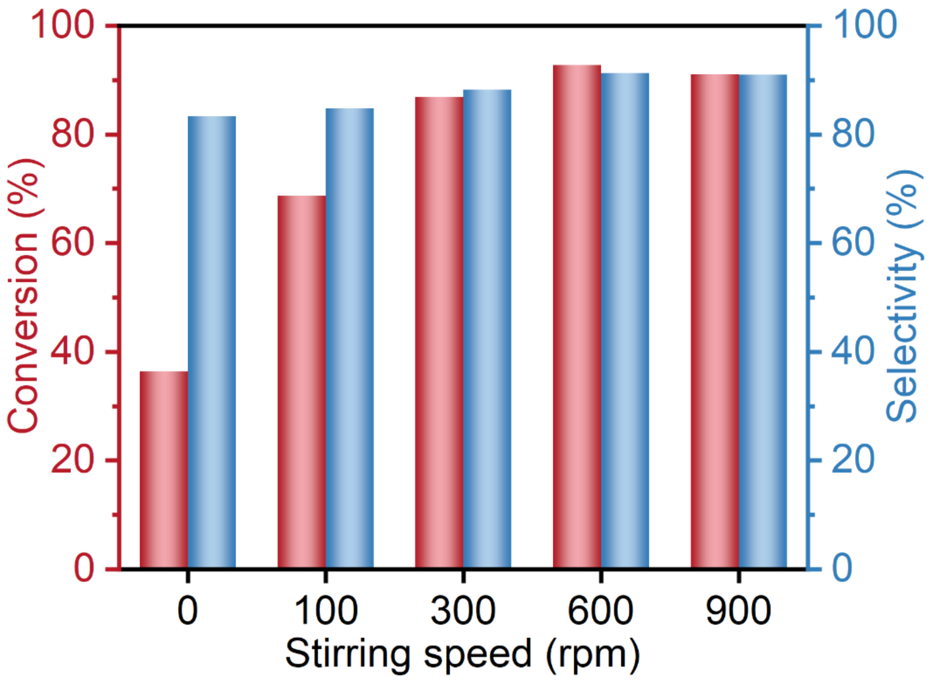

Figure S15. The optimization of the stirring speed for the Mukaiyama epoxidation of 1-undecene catalyzed by Cu/MCM-22.

This figure illustrated the effect of stirring speed on the conversion (red bars) and selectivity (blue bars) in the Mukaiyama epoxidation of 1-undecene. The conversion of 1-undecene was less than 40% in the absence of stirring, indicating significant diffusion limitations. Increasing the stirring speed to 600 rpm enhanced the conversion, likely due to improved mixing and reduced mass transfer resistance. However, at 900 rpm, further improvements were no longer observed, suggesting that the reaction had shifted from being diffusion-limited to being constrained by the intrinsic catalytic activity. Based on these findings, a stirring speed of 600 rpm was determined to be optimal. Reaction conditions: 5.0 mL of acetonitrile, 1.0 mmol of 1-undecene, 5.0 mmol of benzaldehyde, O_2 balloon, 20.0 mg of catalyst, 60 ℃, 6 hours. RPM (revolutions per minute) denotes the stirring speed.

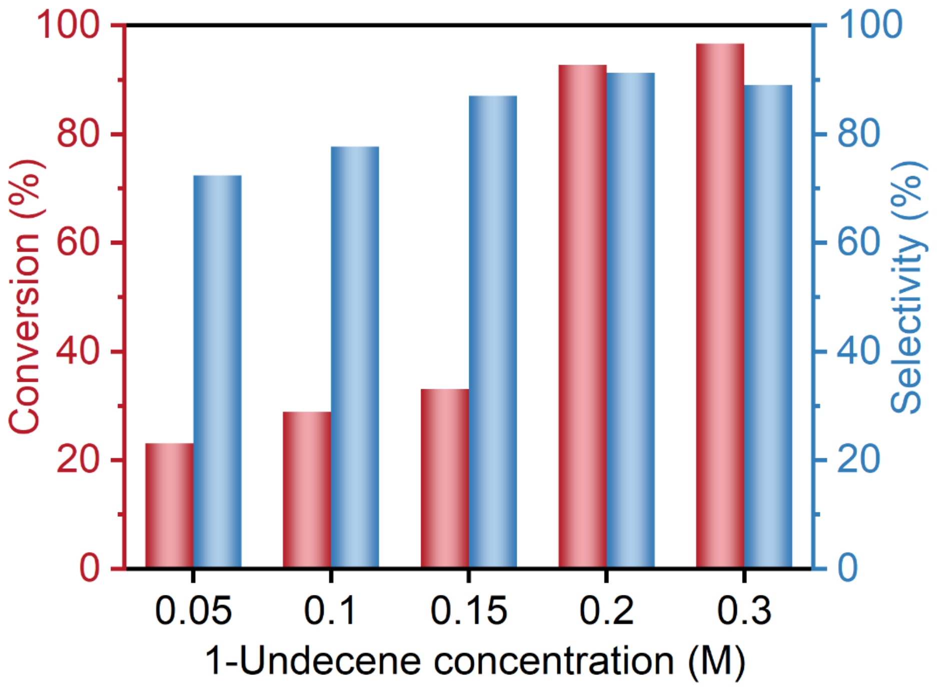

Figure S16. The optimization of 1-undecene concentration for the Mukaiyama epoxidation of 1-undecene catalyzed by Cu/MCM-22.

This figure illustrated the effect of 1-undecene concentration on the conversion (red bars) and selectivity (blue bars) in the Mukaiyama epoxidation of 1-undecene. At a fixed stirring speed, the performance increased with concentration at low levels, consistent with kinetic control, but plateaued at higher concentrations as mass-transfer limitations were reached. Based on these findings, a concentration of 0.2 M 1-undecene (corresponding to 5.0 mL of acetonitrile) was identified as the optimal condition. Reaction conditions: 1.0 mmol of 1-undecene, 5.0 mmol of benzaldehyde, O_2 balloon, 20.0 mg of catalyst, 60 ℃, 600 rpm,6 hours.

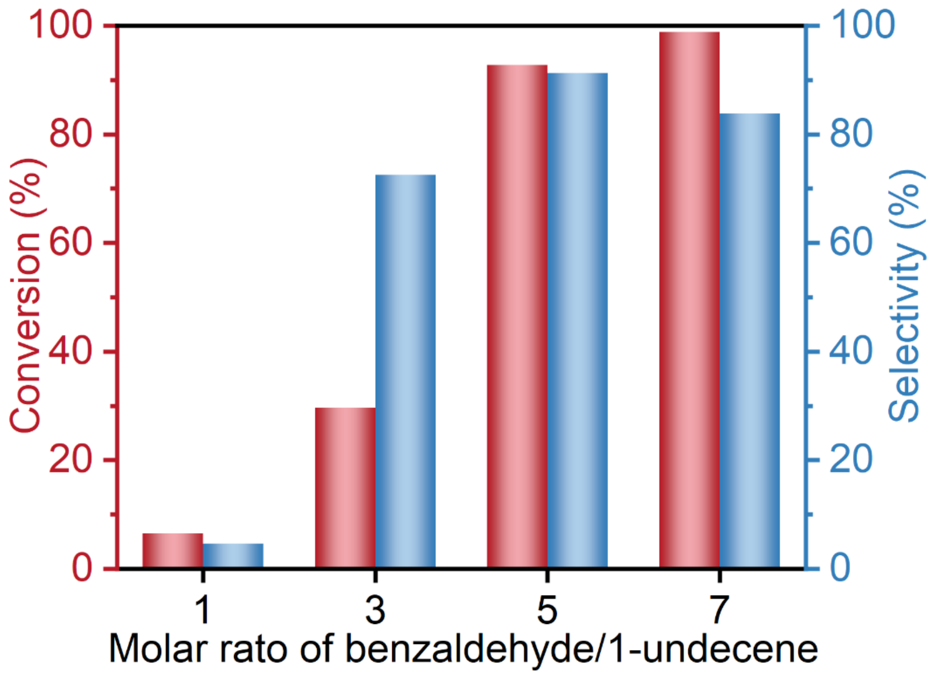

Figure S17. The optimization of the equivalent of benzaldehyde for the Mukaiyama epoxidation of 1-undecene catalyzed by Cu/MCM-22.

This figure illustrated the effect of the equivalent of benzaldehyde on the conversion (red bars) and selectivity (blue bars) in the Mukaiyama epoxidation of 1-undecene. When 1 equivalent of benzaldehyde was used, both the conversion rate and selectivity were relatively low, likely due to insufficient benzaldehyde to drive the reaction. Increasing the equivalent to 3 resulted in significant improvements in both metrics, suggesting a more favorable reactant ratio. The selectivity reached its peak at 5 equivalents, indicating that this amount of benzaldehyde was sufficient for complete reaction with 1-undecene, without excess that could lead to side reactions. However, when the equivalent was increased to 7, the conversion continued to rise, but selectivity began to decline, possibly due to excess benzaldehyde promoting side reactions or reducing catalyst efficiency. Based on these results, 5 equivalents of benzaldehyde was identified as optimal. Reaction conditions: 5.0 mL of acetonitrile, 1.0 mmol of 1-undecene, O_2 balloon, 20.0 mg of catalyst, 60 ℃, 600 rpm, 6 hours.

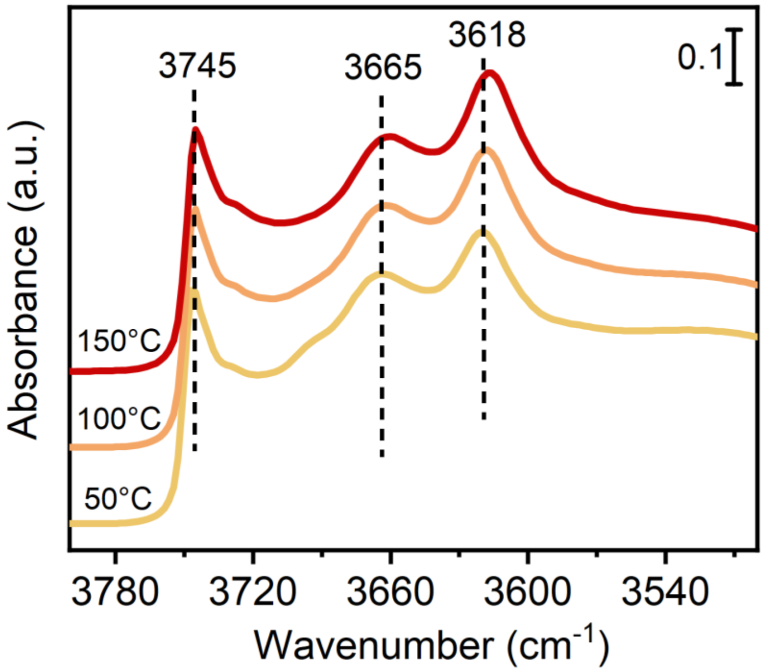

Figure S18. Hydroxyl infrared spectra of Cu/MCM-22-Im measured at 50, 100, and 150 ℃.

No Cu–OH characteristic peaks were observed in the spectra, in contrast to the ion-exchanged Cu/MCM-22 (Figure 1D, observed at 100 ℃), indicating the absence of isolated Cu–OH active sites in the impregnated samples.

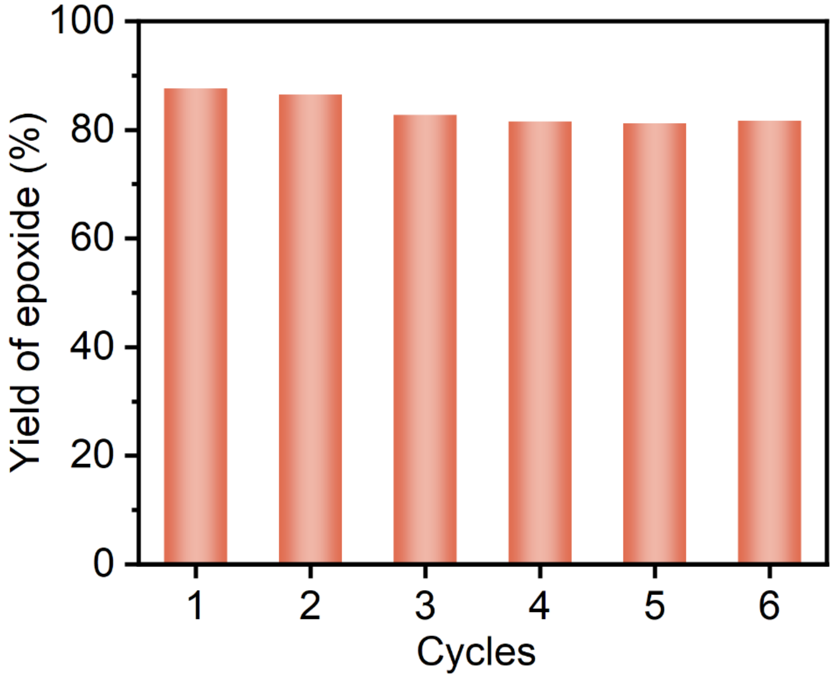

Figure S19. Recyclability of Cu/MCM-22 tested over 6 cycles in the Mukaiyama epoxidation of 1-undecene.

This figure illustrated that the Cu/MCM-22 catalyst exhibited excellent catalytic stability, maintaining a yield of over 80% across 6 cycles. Reaction conditions: 5.0 mL of acetonitrile, 1.0 mmol of 1-undecene, 5.0 mmol of benzaldehyde, O_2 balloon, 20.0 mg of catalyst, 60 ℃, 8 hours, 600 rpm.

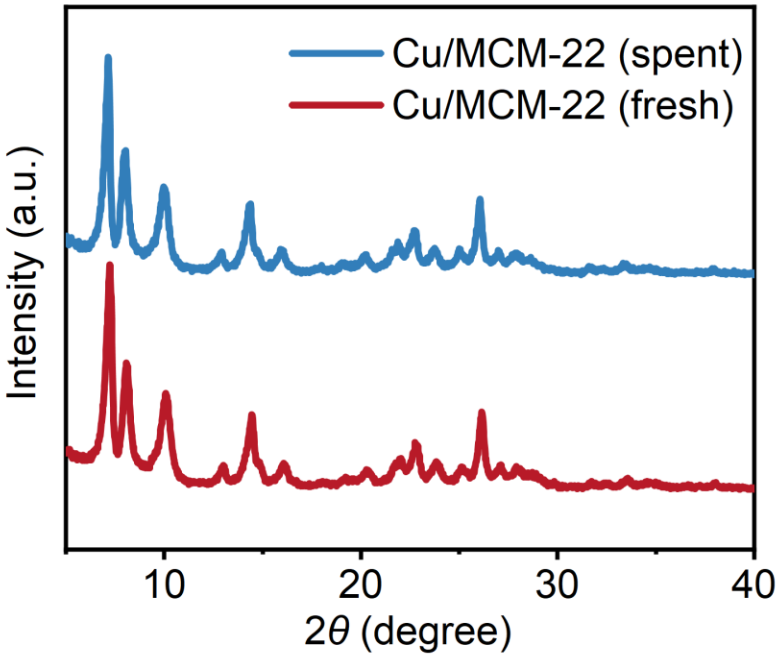

Figure S20. XRD patterns of the spent Cu/MCM-22 and the fresh Cu/MCM-22.

The XRD patterns of both fresh and spent Cu/MCM-22 catalysts showed no significant differences, indicating that the reaction process did not alter the crystalline structure or morphology of the catalyst.

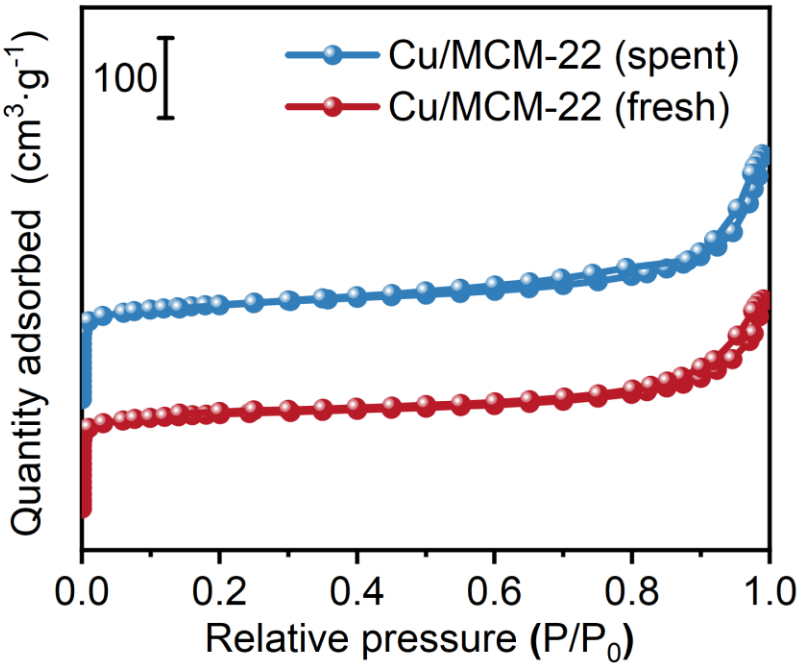

Figure S21. N_2 physisorption isotherms of the spent Cu/MCM-22 and the fresh Cu/MCM-22.

The N_2 adsorption-desorption isotherms of Cu/MCM-22 samples showed no significant differences before and after the reaction, indicating that the reaction process did not affect the textural or porosity properties of Cu/MCM-22.

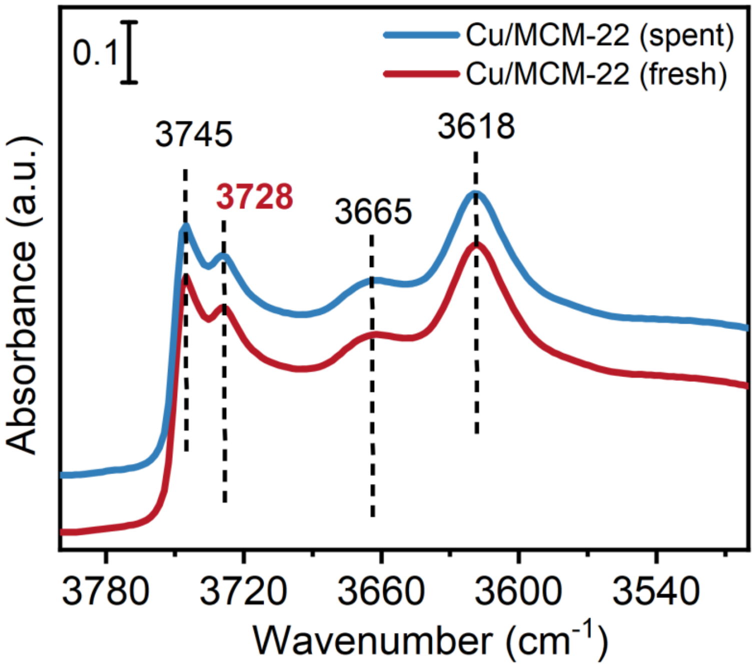

Figure S22. Hydroxyl infrared spectra of Cu/MCM-22 before (fresh) and after six reaction cycles (spent).

The Cu–OH characteristic peak at 3728 cm^{-1} remains nearly unchanged, indicating the stability of the isolated Cu–OH active sites during repeated catalytic cycles.

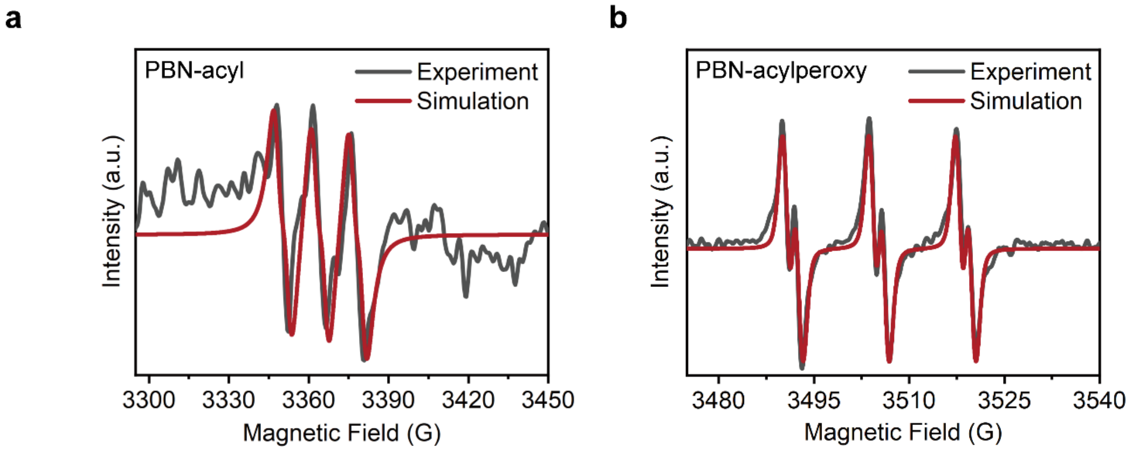

Figure S23. EPR X-band spin-trapping spectra of acyl and acylperoxy radicals captured by PBN.

Comparison of the (a) anaerobic and (b) aerobic EPR spectra of PBN-derived adducts from a post-reaction mixture of benzaldehyde. The EPR spectrum under anaerobic condition showed acyl radicals, while under aerobic condition, acylperoxy radicals were observed.

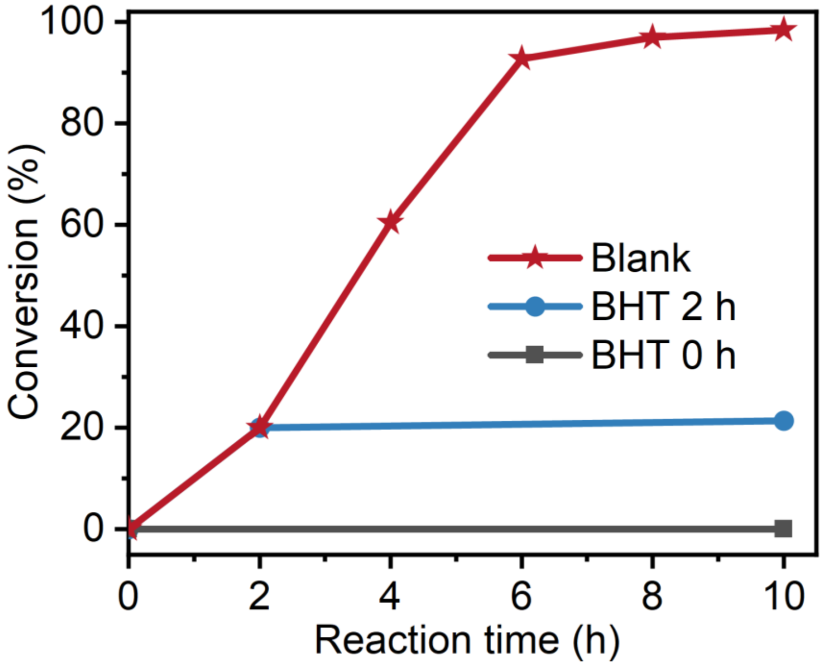

Figure S24. Effect of radical scavengers on the Mukaiyama epoxidation of 1-undecene.

The reaction was suppressed by the addition of the radical scavenger BHT, confirming the free-radical nature of the process and identifying acylperoxy radicals as the primary oxidants.

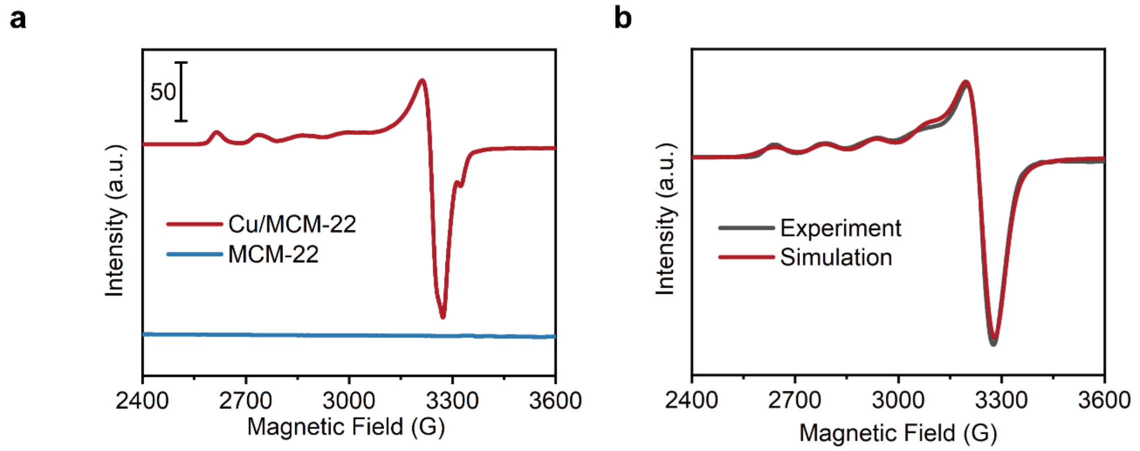

Figure S25. EPR spectra of solid samples for Cu/MCM-22 and MCM-22 testing under 100 K.

As shown in the figures, (a) the peak at 2500-3400 G could be clearly observed in the Cu/MCM-22 compared with MCM-22, which proved that the Cu in the MCM-22 was the Cu^{2+} through simulation (b).

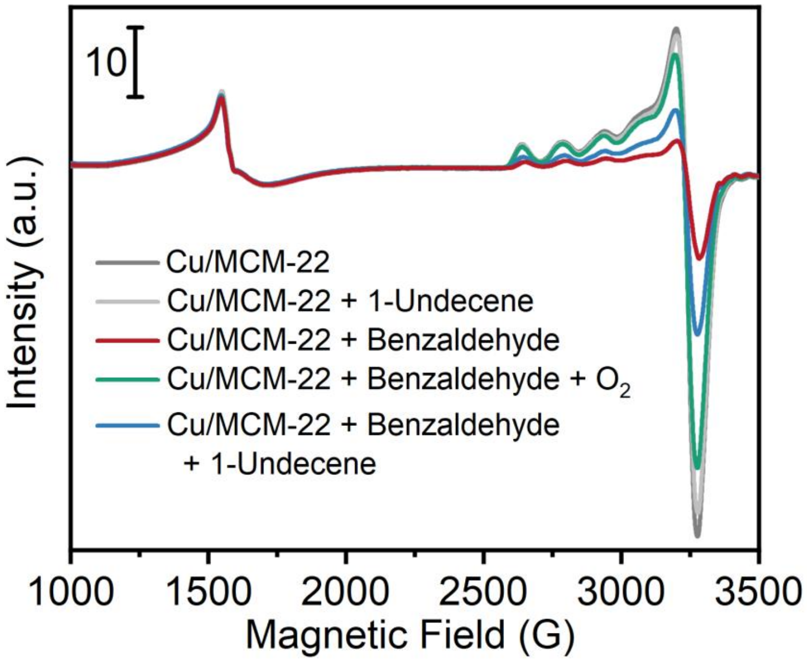

Figure S26. Operando EPR spectra of a treated reaction mixture testing under 100 K.

The signal intensity changes of divalent copper (Cu^{2+}) in the Cu/MCM-22 catalyst under different conditions were investigated using low-temperature EPR spectroscopy. The vertical axis in the figure represents the EPR signal intensity of Cu^{2+}. The results showed that the Cu^{2+} signal in the control group (containing only Cu/MCM-22) was the strongest, indicating that Cu^{2+} in the catalyst remained in a more active state in the absence of reactants. The signal intensity remained largely unchanged after the addition of 1-undecene, but when benzaldehyde was introduced, the Cu^{2+} signal gradually decreased. This suggested that benzaldehyde may interact with Cu^{2+}, altering its electronic structure. After the benzaldehyde-exposed sample was placed in an oxygen atmosphere, the signal intensity partially recovered, indicating a reversible transformation in the valence state of Cu species during the interaction with benzaldehyde. The peak at 1500 G was the peak of the test tube as a reference.

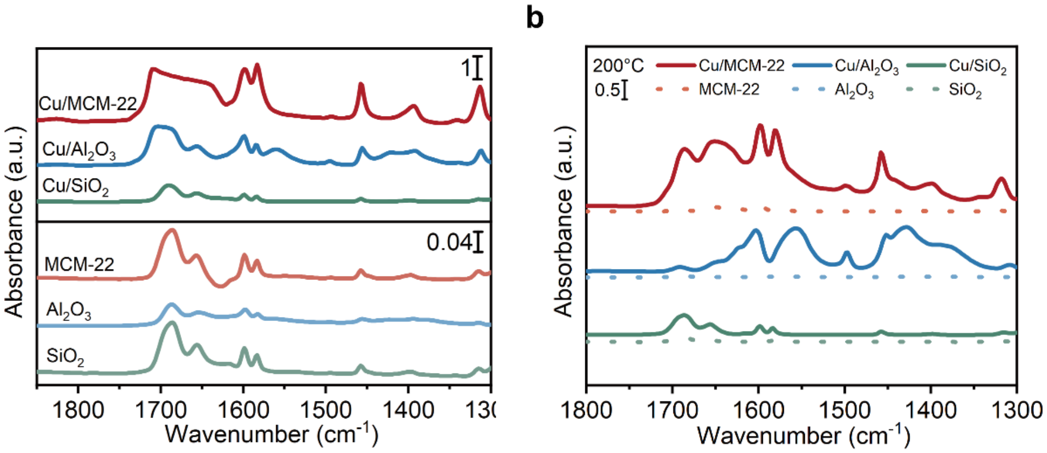

Figure S27. FT-IR spectra of catalysts upon benzaldehyde adsorption.

(a) Spectra after saturation of benzaldehyde adsorption, where Cu-loaded catalysts show much stronger bands than Cu-free samples, indicating that Cu sites are the primary adsorption centers. (b) Spectra during desorption at 200 ℃, where the adsorption peaks of Cu-free catalysts nearly disappeared, while Cu/MCM-22 retained much stronger signals than Cu/Al_2O_3 and Cu/SiO_2, demonstrating that Cu sites in MCM-22 are more favorable for benzaldehyde adsorption.

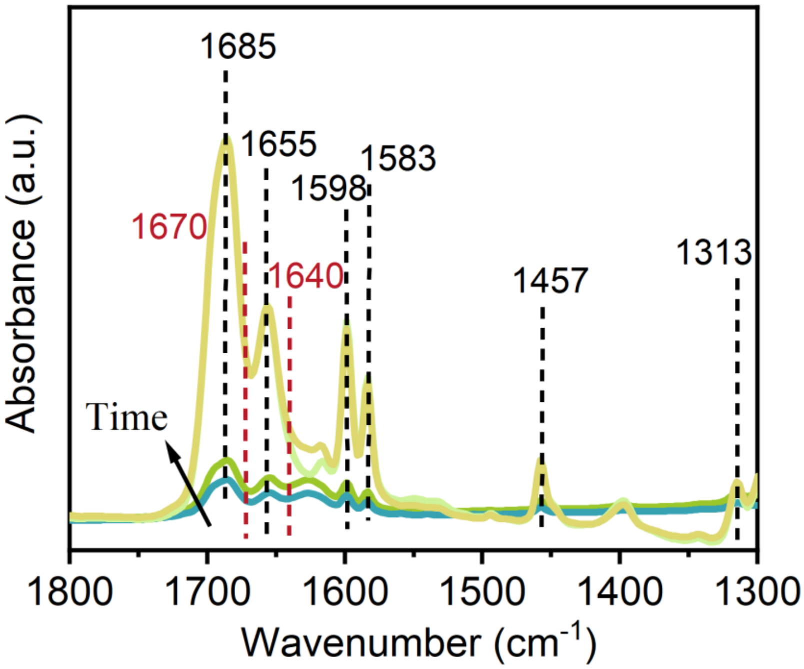

Figure S28. FT-IR spectra of SiO_2 sample upon benzaldehyde adsorption with changes in adsorption time.

The figure showed that there is no water peak at 1640 and 1670 cm^{-1}, emphasizing no water in benzaldehyde reagent. The adsorption time gradually increases from green to yellow.

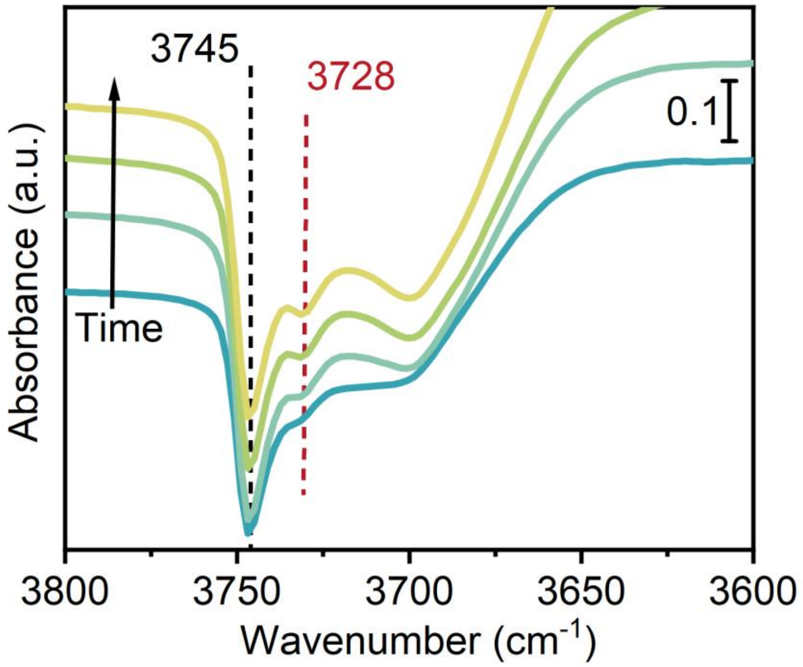

Figure S29. FT-IR spectra in the hydroxyl region, derived from the same dataset as Figure 3D. The Cu-OH characteristic band (3728 cm^{-1}) gradually evolved with increasing benzaldehyde adsorption time. The adsorption time gradually increases from green to yellow.

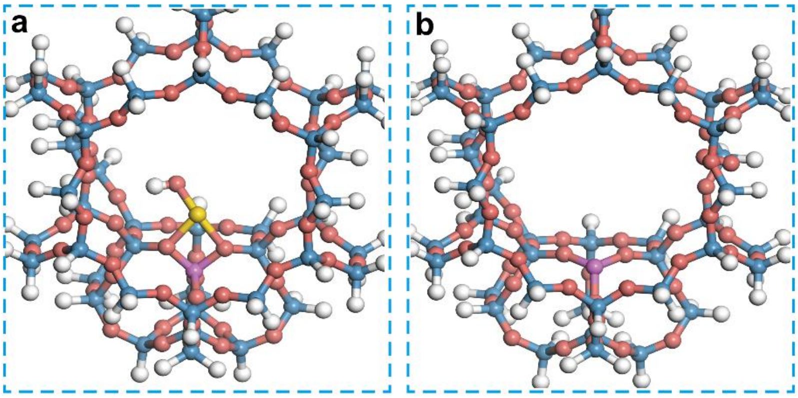

Figure S30. Theoretical models of metal active sites.

(a) Isolated Cu-OH site inside the MCM-22 zeolite. (b) Al site in the MCM-22 zeolite as controls. The Cu, Al, Si, O and H atoms are shown in yellow, purple, blue, red and white, respectively.

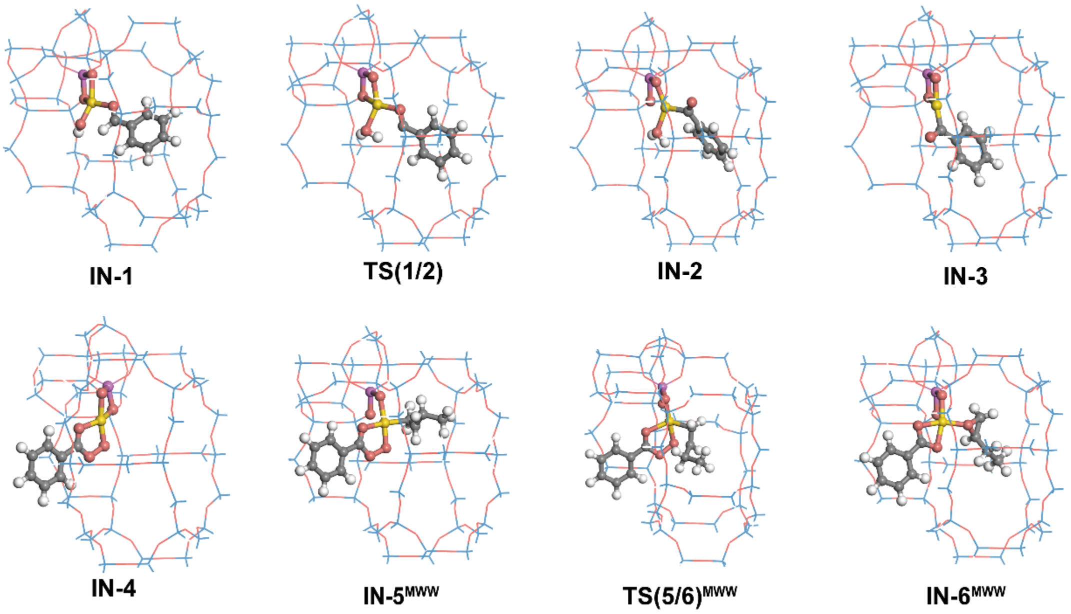

Figure S31. Theoretical structures of intermediates for 1-butene Mukaiyama epoxidation on Cu/MCM-22 model.

The Cu, Al, Si, O, C and H atoms are shown in yellow, purple, blue, red, grey and white, respectively.

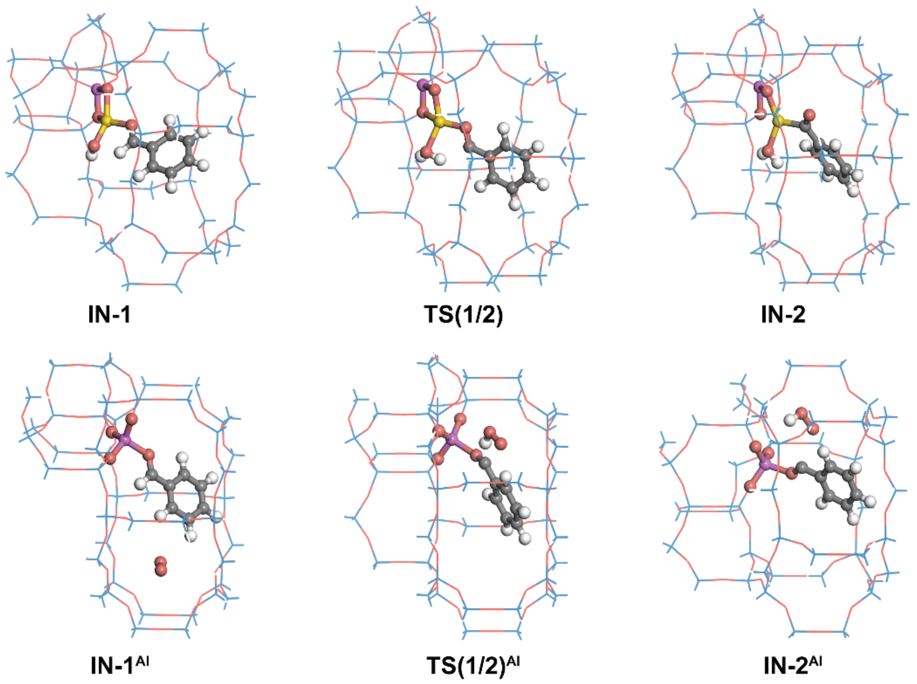

Figure S32. Theoretical structures of intermediates for the activation of benzaldehyde. "IN-1, TS(1/2), IN-2" and "IN-1^{Al}, TS(1/2)^{Al}, IN-2^{Al}" refer to the intermediates on Cu-OH sites and Al sites, respectively. The Cu, Al, Si, O, C and H atoms are shown in yellow, purple, blue, red, grey and white, respectively.

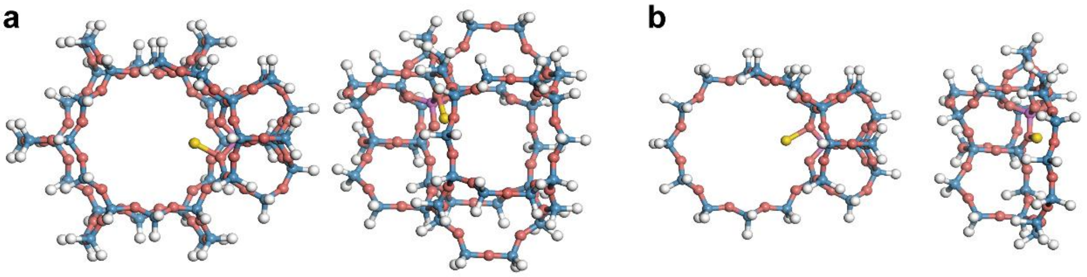

Figure S33. Theoretical models of MWW and Meso-MWW.

(a) Model cluster of MWW zeolite. (b) Model cluster of Meso-MWW. The Cu, Al, Si, O, C and H atoms are shown in yellow, purple, blue, red, grey and white, respectively.

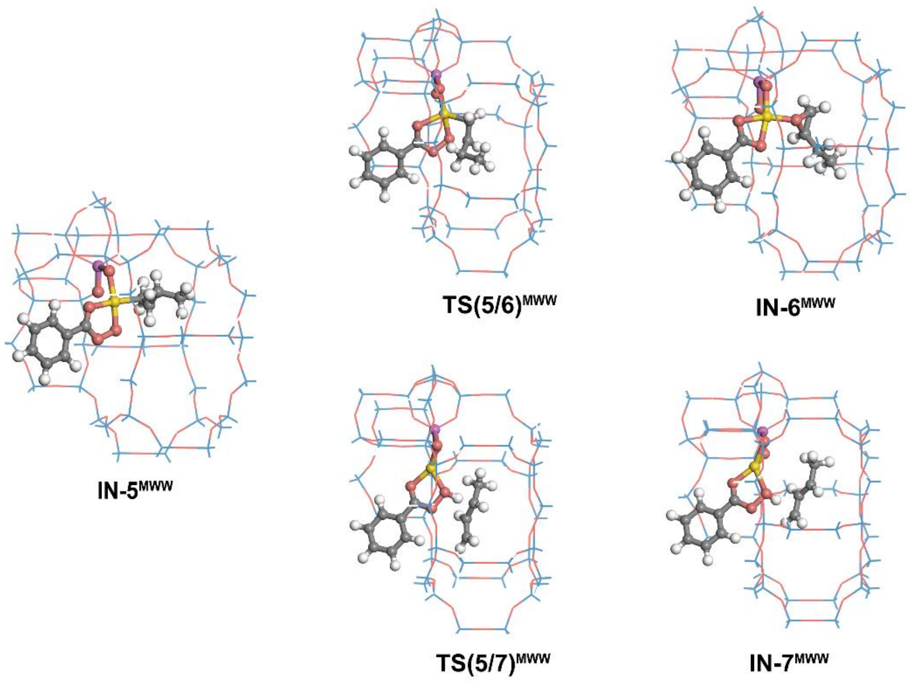

Figure S34. Theoretical structures of intermediates for 1-butene epoxidation on MWW model.

The Cu, Al, Si, O, C and H atoms are shown in yellow, purple, blue, red, grey and white, respectively.

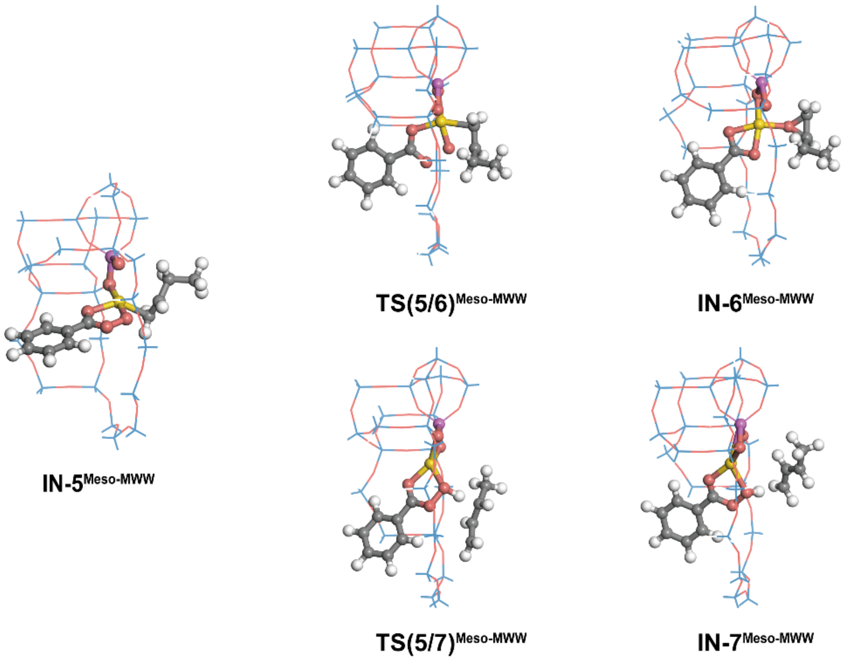

Figure S35. Theoretical structures of intermediates for 1-butene epoxidation on Meso-MWW model.

The Cu, Al, Si, O, C and H atoms are shown in yellow, purple, blue, red, grey and white, respectively.

Table S1. Calculated dimensions of MCM-22 and intermediates in all three pathways, taking 1-undecene as an example.

Zeolite/Intermediates	Dimensions (Å)
MCM-22	7.1×7.1
intermediate-A	23.7×5.9×4.8
intermediate-B	21.6×8.3×5.4
intermediate-D	17.3×7.4×4.3

Intermediate-A, B, and D correspond to Scheme S1 (R^1 = phenyl; R^2 = n-octyl). Reactants diffuse through 10-MR channels of MCM-22 and undergo transformation at 12-MR (7.1 × 7.1 Å), where steric constraints suppress formation of B and D.

Table S2. Catalytic performance of Mukaiyama epoxidation of 1-undecene catalyzed by a series of Na-type silicoaluminate zeolite samples.

Samples	Conversion of 1-undecene (%)	Selectivity of epoxide (%)	Yield of epoxide (%)
MCM-22	20.5	82.2	16.9
Beta	29.5	76.2	22.5
NaY	21.0	70.0	14.5

Reaction conditions: 5.0 mL of acetonitrile, 1.0 mmol of 1-undecene, 5.0 mmol of benzaldehyde, O_2 balloon, 20.0 mg of catalyst, 60 ℃, 6 hours, 600 rpm.

Table S3. Catalytic performance of Mukaiyama epoxidation of 1-undecene catalyzed by Cu/MCM-22, Cu/Beta and Cu/Y samples. The error bars of conversion of 1-undecene and selectivity for 1,2-epoxyundecane are calculated based on three independent measurements.

Samples	Conversion of 1-undecene (%)				Selectivity of epoxide (%)			
	Run1	Run2	Run3	Average ± SD	Run1	Run2	Run3	Average ± SD
Cu/MCM-22	92.7	93.9	93.4	93.3 ±0.6	91.3	92.5	92.9	92.2 ±0.8
Cu/Beta	90.7	91.0	90.0	90.5 ±0.5	86.5	85.7	87.5	86.5 ±0.9
Cu/Y	87.0	87.4	86.1	86.8 ±0.6	84.3	87.4	82.2	84.6 ±2.6

Reaction conditions: 5.0 mL of acetonitrile, 1.0 mmol of 1-undecene, 5.0 mmol of benzaldehyde, O_2 balloon, 20.0 mg of catalyst, 60 ℃, 6 hours, 600 rpm.

Table S4. Physical properties and elemental composition of samples.

Samples	Surface area (m^2 g^{-1})			Micropore volume[d] (m^3 g^{-1})	Pore size (nm)	Cu (wt.%)
	Total[a]	External[b]	Micro[c]			
Cu/MCM-22	474	69	405	0.18	0.52	0.51
MCM-22	499	103	396	0.18	0.51	-
Cu/Al_2O_3	123	100	23	0.01	23.74	0.49
Al_2O_3	141	126	15	0.01	20.37	-
Cu/SiO_2	499	-	-	-	6.13	0.52
SiO_2	515	-	-	-	5.98	-

[a] From BET method. [b, c, d] From t-plot method.

Table S5. Comparison of catalytic performance of epoxidation of 1-undecene under air and oxygen condition using Cu/MCM-22 catalyst.

	Conversion of 1-undecene (%)	Selectivity of epoxide (%)	Yield of epoxide (%)
Air	21.6	94.6	20.4
O_2	93.3	92.2	86.0

Reaction conditions: 5.0 mL of acetonitrile, 1.0 mmol of 1-undecene, 5.0 mmol of benzaldehyde, O_2 balloon, 20.0 mg of catalyst, 60 ℃, 6 hours, 600 rpm.

Table S6. Catalytic performance of Mukaiyama epoxidation of 1-undecene catalyzed by unconfined systems and confined zeolites systems.

Samples	Conversion of 1-undecene (%)	Selectivity of epoxide (%)	Yield of epoxide (%)	Aldehyde coupling efficiency (%)	Conversion of benzaldehyde (%)
Cu/MCM-22	93.3	92.2	86.0	53.4	32.2
MCM-22	20.5	82.2	16.9	-	-
Cu/Al_2O_3	80.1	65.2	52.2	29.6	35.3
Al_2O_3	20.2	61.9	12.5	-	-
Cu/SiO_2	76.8	61.8	47.5	25.6	37.1
SiO_2	15.6	56.1	8.8	-	-
Blank	18.9	56.3	10.7	-	-

Reaction conditions: 5.0 mL of acetonitrile, 1.0 mmol of 1-undecene, 5.0 mmol of benzaldehyde, O_2 balloon, 20.0 mg of catalyst, 60 ℃, 6 hours, 600 rpm.

Table S7. Catalytic performance of Mukaiyama epoxidation of 1-undecene catalyzed by Cu/MCM-22 and Cu/MCM-22-Im samples.

Samples	Conversion of 1-undecene (%)	Selectivity of epoxide (%)	Yield of epoxide (%)
Cu/MCM-22	93.3	92.2	86.0
Cu/MCM-22-Im	83.8	92.1	77.3

Reaction conditions: 5.0 mL of acetonitrile, 1.0 mmol of 1-undecene, 5.0 mmol of benzaldehyde, O_2 balloon, 20.0 mg of catalyst, 60 ℃, 6 hours, 600 rpm.

Table S8. Catalytic performance of Mukaiyama epoxidation of 1-undecene catalyzed by Cu/MCM-22 and modifier-Cu/MCM-22 samples.

Samples	Conversion of 1-undecene (%)	Selectivity of epoxide (%)	Yield of epoxide (%)	Aldehyde coupling efficiency (%)	Conversion of benzaldehyde (%)
Cu/MCM-22	97.0	90.3	87.6	43.0	40.7
Cu_{TPA}/MCM-22	79.0	99.9	78.9	50.0	31.6
Cu_{PQ}/MCM-22	79.0	99.1	78.3	60.5	25.9

Reaction conditions: 5.0 mL of acetonitrile, 1.0 mmol of 1-undecene, 5.0 mmol of benzaldehyde, O_2 balloon, 20.0 mg of catalyst, 60 ℃, 8 hours, 600 rpm.

Table S9. Overall observed components carbon balance based on the 1-undecene for epoxidation of 1-undecene over Cu/MCM-22 and modifier-Cu/MCM-22 samples.

Samples	C_{feed}	C_{output}			Carbon balance (%)
	$C_{1\text{-undecene}}$[a]	$C_{1\text{-undecene}}$[b]	$C_{1,2\text{-epoxyundecane}}$[c]	$C_{\text{allylic byproducts}}$[d]	
Cu/MCM-22	11	0.32	9.62	0.84	98.0
Cu_{TPA}/MCM-22	11	2.28	8.55	0.13	99.6
Cu_{PQ}/MCM-22	11	2.31	8.60	0.08	99.8

[a] The mole of carbon in the feeding of 1-undecene.

[b] The mole of carbon in the unconverted 1-undecene.

[c] The mole of carbon in the generated 1,2-epoxyundecane.

[d] The mole of carbon in the generated allylic byproducts.

Table S10. Benzaldehyde mass balance for epoxidation of 1-undecene over Cu/MCM-22 sample.

Sample	n_{feed}	n_{output}			Mass balance (%)	Benzaldehyde byproducts (%)
	$n_{benzaldehyde}$[a]	$n_{benzaldehyde}$[b]	$n_{benzoic\ acid}$[c]	$n_{byproducts}$[d]		
Cu/MCM-22	5.0	3.0	1.8	0.1	98.0	5.3

[a] The mole of feeding benzaldehyde.

[b] The mole of unconverted benzaldehyde.

[c] The mole of generated benzoic acid.

[d] The mole of generated byproducts.

Table S11. Comparison of the catalytic performances of Cu/MCM-22, Cu_{PQ}/MCM-22 with various catalysts in long-chain LAOs epoxidation.

Olefins	Catalysts	TOF (h^{-1})	Yield of epoxide (%)	Ref.
1-decene	Sn-Beta	42.8	84.4	[3]
1-decene	CuO/Al_2O_3	16.6	99.0	[4]
1-decene	CoO_x/S-1	5.3	26.9	[5]
1-octene	Mn/SiO_2	21.2	25.3	[6]
1-heptene	Cu-MOF	10.4	50.0	[7]
1-decene	$Ru-SiO_2$	0.3	58.0	[8]
1-decene	Co/HMS	3.7	86.5	[9]
1-octene	Sn_1/V_o-CuO	4.0	92.9	[10]
1-undecene	Cu_{PQ}/MCM-22	77.2	78.3	This work
1-undecene	Cu/MCM-22	87.8	84.6	This work

Table S12. Catalytic performance of Mukaiyama epoxidation of different olefins catalyzed by Cu/MCM-22 sample.

Olefins	Conversion of olefin (%)	Selectivity of epoxide (%)	Yield of epoxide (%)
1-undecene	97.0	90.3	87.6
1-decene	93.9	88.3	83.0
1-nonene	90.9	85.2	77.5
1-octene	97.6	84.5	82.5
1-heptene	95.4	82.7	78.9
cyclooctene	3.8	13.3	0.51

Reaction conditions: 5.0 mL of acetonitrile, 1.0 mmol of olefin, 5.0 mmol of benzaldehyde, O_2 balloon, 20.0 mg of catalyst, 60 ℃, 8 hours, 600 rpm.

Table S13. Summary of simulation parameters for EPR spectra in spin-trapping experiment.

Radical	***g-value***	A^N/G	A^H/G	**lw/mT**
PBN-acyl	2.092	14.7	3.7	0.1, 0.7
PBN-acylperoxy	2.008	13.7	1.8	0.15, 0.08

lw is the homogeneous Lorentzian linewidth; the linewidth defines the FWHM (full width at half height, in mT) of the absorption Gaussian (first number) and Lorentzian (second number) broadening of the lines in the spectrum.

Table S14. Catalytic performance of Mukaiyama epoxidation of 1-undecene catalyzed by Cu/MCM-22 catalyst with radical scavengers.

Reaction time (h)	2	4	6	8	10	10*	10**
Conversion of 1-undecene (%)	20.0	60.4	92.7	97.0	98.4	0	21.4

*: reaction was conducted for 0 hour before the addition of radical scavenger (BHT: 2,6-ditert-butyl-4-methylphenol), and then extended to 10 hours. **: reaction was conducted for 2 hours before the addition of BHT, and then extended to 10 hours.

Table S15. Summary of simulation parameters for EPR spectrum of Cu/MCM-22.

Sample	g_{xy}	g_z	A^{xy} (G)	A^{z} (G)	**lw/mT**
Cu/MCM-22	2.069	2.347	/	143	0.1, 0.7

lw is the homogeneous Lorentzian linewidth; the linewidth defines the FWHM (full width at half height, in mT) of the absorption Gaussian (first number) and Lorentzian (second number) broadening of the lines in the spectrum.

SI References

1. Stoll S, Schweiger A. EasySpin, a comprehensive software package for spectral simulation and analysis in EPR. *J Magn Reson* 2006; **178**: 42–55.
2. Chen Y, Wang S, Wei Z *et al.* Unraveling the relationship between zeolite structure and MTO product distribution by theoretical study of the reaction mechanism. *J Phys Chem C* 2021; **125**: 26472–83.
3. Zhou Z, Zhang K, He P *et al.* Reactive intermediate confinement in Beta zeolites for the efficient aerobic epoxidation of α-olefins. *Angew Chem Int Ed* 2025; **64**: e202419900.
4. Cao J, Zhou Z, Zhang M *et al.* Tuning the electronic properties of supported Cu catalysts for efficient epoxidation of long-chain α-olefins. *Fuel* 2023; **342**: 127829.
5. Li C, Pu N, Huang K *et al.* Highly efficient and robust aerobic co-oxidation of olefins and aldehydes over CoO_x dispersed within hierarchical silicalite-1 zeolites. *Green Chem* 2022; **24**: 6200–14.
6. Muratsugu S, Weng Z, Tada M. Surface functionalization of supported Mn clusters to produce robust Mn catalysts for selective epoxidation. *ACS Catal* 2013; **3**: 2020–30.
7. Feng X, Song Y, Chen JS *et al.* Rational construction of an artificial binuclear copper monooxygenase in a metal-organic framework. *J Am Chem Soc* 2021; **143**: 1107–18.
8. Tada M, Muratsugu S, Kinoshita M *et al.* Alternative selective oxidation pathways for aldehyde oxidation and alkene epoxidation on a SiO_2-supported Ru−monomer complex catalyst. *J Am Chem Soc* 2010; **132**: 713–24.
9. Shi Z-Q, Jiao L-X, Sun J *et al.* Cobalt nanoparticles in hollow mesoporous spheres as a highly efficient and rapid magnetically separable catalyst for selective epoxidation of styrene with molecular oxygen. *RSC Adv* 2014; **4**: 47–53.
10. Zhang M, Qin G, Li P *et al.* Enhanced catalytic performance of Sn single-atom doped CuO with oxygen vacancies for efficient epoxidation of α-olefins. *Molecules* 2025; **30**: 1042.
